# Confidence without consensus? Human versus AI abstracts in urology

**DOI:** 10.1007/s00345-026-06872-6

**Published:** 2026-10-16

**Authors:** Sholem Hack, Shlomo Resnik, Yoram Dekel, Shlomi Moshe, Daniel Noy, Mohammad Shalabi, Ari Frajewicki, Omri Zonenfeld, Sobhi Khoury, Israel Benzekry, Yazeed Barghouthy, Maharan Kabha

**Affiliations:** 1 City St. George’s University London School of Medicine, Program Delivered by University of Nicosia, Chaim Sheba Medical Center, Ramat Gan, Israel; 2 St. George’s University School of Medicine, St. George, West Indies Grenada; 3 Department of Urology, Carmel Medical Center, Haifa, Israel; 4 Service d’Urologie, AP-HP Centre, Hôpital Européen Georges Pompidou, Université de Paris, Paris, France; 5 Herta and Paul Amir School of Medicine, University of Haifa, Haifa, Israel

**Keywords:** Abstract quality, Authorship detection, Large language models, Urology, Peer review, Scientific publishing

## Abstract

**Objective:**

This study asked whether urologists can tell human-written abstracts apart from artificial intelligence (AI)-written abstracts when both describe the same study, and whether the answer depends on which AI model did the writing.

**Methods:**

Fifteen published urology studies were selected. For each study, the original human-written abstract was paired with three AI-written abstracts covering the same material, produced by Claude Sonnet 4.6, GPT-5.5, and Gemini 3 Flash using identical instructions and each model’s ordinary consumer settings. Because all four versions described the same study, the underlying data were held constant, and the versions differed in how that material was written and presented. The resulting 60 abstracts were stripped of identifying information, and eight urologists read all of them without knowing which was which (480 judgments in total). For each abstract, reviewers stated whether they believed it was written by a human or by AI, rated how confident they were in that judgment, and rated the abstract’s quality and credibility on 5-point scales.

**Results:**

Reviewers correctly identified AI-written abstracts 49.3% of the time, approximately 50%, compared with 62.4% for human-written abstracts (*p* = .011). Results depended heavily on which model wrote the text (*p* = .003). Abstracts written by Claude were recognized as AI only 40.0% of the time, significantly less often than human abstracts were correctly identified (*p* = .003) and less often than GPT abstracts (56.8%, *p* = .038); Gemini fell between the two at 50.9%. Quality ratings differed only for GPT, which scored lower than human abstracts (*p* = .046; omnibus *p* = .030); credibility ratings did not differ between sources at all (*p* = .577). Reviewers who felt confident were no more likely to be correct (*p* = .749), and reviewers seldom agreed with one another about which abstracts were AI-written (Fleiss’ κ = −0.009).

**Conclusion:**

Urologists could not reliably recognize AI-written urology abstracts, and the difficulty came mainly from one model rather than from AI writing in general. How good an abstract appeared had little to do with whether its authorship was correctly identified, and neither reviewer confidence nor agreement between reviewers gave any warning. Reading alone is therefore not a dependable way to detect undisclosed AI authorship.

**Supplementary Information:**

The online version contains supplementary material available at https://doi.org/10.1007/s00345-026-06872-6.

## Introduction

Large language models (LLMs) have moved rapidly from novelty to routine infrastructure across medicine, with documented applications spanning clinical decision support, patient communication, medical education, and the drafting of scientific text [1–4]. Their fluency in academic English makes them attractive to authors, but that same fluency raises a persistent concern: undisclosed AI text may be hard to separate from writing by the investigators who actually collected the data, and may carry unverifiable content into the literature [5]. 

Early work addressed this concern empirically. Gao et al. found that blinded reviewers could detect ChatGPT-generated biomedical abstracts with reasonable but imperfect accuracy, relying largely on impressions of vagueness and formulaic phrasing to do so [6, 7]. Subsequent studies across specialties have produced a strikingly wide range of results, from essentially chance-level performance among experienced editorial board reviewers in a blinded essay contest [8], to intermediate accuracy in randomized survey designs [9] and in gynecology and urogynecology [10], to near-total failure among orthopedic reviewers and commercial AI-detection software alike [11]. Comparable heterogeneity has been reported in educational settings [12]. 

Two recent findings sharpen the question. A contemporaneous otolaryngology study using a closely comparable design reported overall recognition accuracy of 44.7%, with substantial variation between individual models and with Claude among those most frequently mistaken for human authorship [13]. Outside controlled research settings, AI-generated abstracts submitted to a large national cardiology congress received blinded ratings statistically indistinguishable from human-authored submissions [14]. 

This heterogeneity suggests that the detectability of AI-generated scientific writing is not a fixed property of “AI text” in general, but may depend substantially on which model produced it, on the specialty and genre involved, and on the specific reviewers doing the judging. If that is correct, aggregate statements about whether reviewers can “spot AI” are of limited value, and the more useful question is model-specific.

Urology has particular reasons to ask it. LLMs have already been evaluated within the specialty for generating patient-facing educational material [15], for answering clinical questions relative to consultant urologists [16], for reproducing guideline updates [17], and for drafting sections of systematic reviews [18]. What has not been examined is authorship detection under a design that holds the underlying study constant while varying the writer, human or a specific LLM, so that differences between abstracts arise from how the same material is written and presented rather than from differences in the source studies themselves.

To address this gap, we conducted a blinded evaluation in which each of 15 urology studies was represented by its original human-written abstract and by three abstracts generated from the same source material by three current LLMs under realistic, default consumer conditions. Detection accuracy, perceived quality and credibility, reviewer confidence, and inter-rater agreement were examined to characterize both whether reviewers could identify AI authorship and how reliable that judgment was.

## Methods

This study was designed as a blinded cross-sectional evaluation of abstracts generated by LLMs compared with human-authored abstracts in urology, using a fully crossed, repeated-measures rating structure in which every reviewer evaluated every abstract.

### Study design and materials

Candidate source studies were assembled by the senior author (M.K.), who supplied a pool of approximately 30 recently published urology articles. From this pool, the first author (S.H.) selected 15 studies by convenience sampling, without reference to abstract length, study design, or subspecialty. Eligible articles were original research, systematic reviews, or consensus and guideline documents published in 2025 or 2026 in general urology or closely related subspecialty journals, reporting a structured abstract and containing sufficient Introduction, Methods, Results and Discussion (IMRaD) content to permit independent abstract generation; case reports, editorials, letters, and commentaries were not considered. The final set comprised articles published in *European Urology*, *European Urology Focus*, *The Lancet Oncology*, *World Journal of Urology*, *Journal of Pediatric Urology*, *The Journal of Sexual Medicine*, *Sexual Medicine Reviews*, and the *American Journal of Obstetrics and Gynecology*, and included randomized controlled and prospective trials, systematic reviews and meta-analyses, consensus and guideline summaries, and a retrospective machine-learning cohort, spanning stone disease, uro-oncology, benign prostatic hyperplasia, paediatric urology, andrology and sexual medicine, and female urology. Neither author involved in study selection participated in the reviewer panel, and no reviewer took part in selection.

From these 15 studies, 60 abstracts were compiled: the 15 published human abstracts and one AI-generated abstract per study from each of the three models. Source material provided to the models was limited to the IMRaD content of each study; all identifying information, including manuscript title, author names, affiliations, journal name, and publication details, was removed before input. All four versions of each abstract therefore described the same underlying study and the same body of source data. The models were not, however, asked to rewrite the published abstract, **b**ut generated a new abstract from the blinded manuscript text, independently determining which findings to report, how much numerical detail to retain, what to emphasise, and how to organise the four sections. Differences between versions therefore reflect writing style together with these content-selection and presentation decisions, rather than differences in the underlying study.

Human comparator abstracts were used as published, with no changes to wording, content, or reported data. Because section-heading conventions differ between journals, the headings of the published abstracts were relabelled to match the four-section structure specified in the generation prompt (Background, Methods, Results, Conclusion), so that heading style could not itself indicate an abstract’s origin. No other alterations were made. AI abstracts were additionally constrained to 230–250 words (Supplementary Material 1).

### LLM generation of abstracts

Abstracts were produced using three commercially available LLMs: Claude Sonnet 4.6 (Anthropic), GPT-5.5 (OpenAI), and Gemini 3 Flash (Google), accessed via their standard consumer chat interfaces between 26 and 27 April 2026. No application programming interface access, retrieval-augmented generation, custom system prompts, or non-default temperature or sampling adjustments were used; each model was queried using its default, publicly available consumer configuration in order to reflect realistic, accessible use rather than optimized or fine-tuned output.

For each source study, a standardized prompt (Supplementary Material 2) was used across all three models to ensure consistency of generation conditions. The prompt instructed the model to produce a structured, four-section abstract (Background, Methods, Results, Conclusion) of 230–250 words, using only the numerical data and findings explicitly provided as input, with explicit instructions prohibiting fabrication, inference, or extrapolation of unreported values, and requiring a final internal word-count check prior to output. Each model was queried once per abstract under these conditions; no outputs were regenerated, hand-edited, or selectively curated prior to inclusion in the study set. Outputs were not edited to enforce the word limit, and the models frequently exceeded it: 35 of 45 generated abstracts were longer than 250 words. Abstract length and numerical density by source are reported in Supplementary Table S1.

Each abstract was generated in a new, separate conversation, so that no prior conversational context was carried between source studies. All three models were accessed through paid consumer subscriptions. Web search and browsing were disabled in all three interfaces to prevent any model from retrieving the source article and thereby circumventing blinding. No other configuration options were altered from the defaults presented on opening, and no extended reasoning or “thinking” modes were deliberately enabled. Because consumer interfaces may apply model routing, memory, or tool availability outside the investigator’s control, and because these defaults change over time, exact reproduction of the generation conditions cannot be guaranteed; this is inherent to the realistic-use design and is noted as a limitation. The full set of 60 abstracts, comprising the 15 human comparators and all 45 model-generated abstracts, is provided in Supplementary Material 1.

### Reviewers and procedure

Eight urologists evaluated all 60 abstracts using a structured online survey, yielding a fully crossed, repeated-measures design (8 reviewers × 60 abstracts = 480 total observations). The 60 abstracts were presented in a single randomised sequence, identical for all reviewers, in which the four versions of each source study were separated rather than appearing consecutively. For each abstract, reviewers were asked to (1) judge whether the abstract was written by a human or by AI, selecting from three options (Human / AI / Unsure); (2) rate their confidence in that judgment on a 5-point scale (1 = not confident at all, 5 = extremely confident); (3) rate the abstract on clarity and quality on a 5-point scale (1 = strongly disagree, 5 = strongly agree with “clearly written and good quality”); (4) rate the abstract’s perceived usefulness and credibility on a 5-point scale with the same anchors; and (5) optionally provide free-text comments on the features that informed their Human-versus-AI judgment. Reviewers also reported their level of clinical expertise and their prior frequency of LLM use.

### Outcomes

The primary outcome was authorship classification accuracy, defined as correct versus incorrect identification of true authorship. Secondary outcomes were reviewer-assigned quality and credibility ratings, calibration between self-reported confidence and actual accuracy, the frequency and distribution of “Unsure” judgments, inter-rater agreement, and descriptive thematic review of free-text rationales.

### Statistical analysis

All analyses were performed in R (version 4.6.0). Because every reviewer judged every abstract, each judgment was non-independent in two ways: judgments sharing a reviewer reflect that reviewer’s individual tendencies, and judgments sharing an abstract reflect that abstract’s particular characteristics. Classification accuracy was therefore analyzed using generalized linear mixed-effects models (binomial family, logit link) with crossed random intercepts for reviewer and for abstract, fitted by maximum likelihood using the lme4 package, so that each reviewer had their own baseline propensity to classify correctly and each abstract its own baseline probability of being correctly classified. Abstract source was entered as a fixed effect, specified first as a two-level contrast (human versus AI collapsed across models; Level A) and then as a four-level factor with human-written abstracts as the reference (Level B). The overall effect of source was tested by likelihood ratio test against the corresponding intercept-only model, and estimated marginal means and pairwise contrasts were obtained using the emmeans package and reported as odds ratios with 95% confidence intervals. Because each source study contributed four abstracts, one per source, models were additionally refitted with source study included as a third random intercept, as a sensitivity analysis. Quality and credibility ratings, being ordinal, were analyzed using cumulative link mixed models with the same random-effects structure, fitted using the ordinal package.

Comparisons between individual sources were made only when the overall test of source reached significance, and were corrected for multiple comparisons using the Holm method. “Unsure” responses (14 of 480 judgments, 2.9%) were excluded from the primary accuracy analyses, because an “Unsure” response records the absence of a classification rather than an incorrect one, and treating the two as equivalent conflates declining to judge with judging wrongly. All principal analyses were nonetheless repeated with “Unsure” coded as incorrect, and the frequency and distribution of “Unsure” responses were examined separately by source and by reviewer. To distinguish genuine detection from a simple tendency to guess “human,” the proportion of abstracts judged human-written was also examined descriptively by source.

Abstract characteristics were compared descriptively and, because each source study contributed exactly one abstract per source, tested using repeated-measures analysis of variance blocked on source study. Homogeneity of variance in abstract length was assessed using the Fligner–Killeen test.

Further analyses examined whether reviewer confidence predicted accuracy, how closely reviewers agreed with one another (Fleiss’ kappa for the authorship judgment; intraclass correlation coefficients for the quality and credibility ratings), and whether “Unsure” responses were related to abstract source. Because one reviewer accounted for most “Unsure” responses, all accuracy analyses were repeated with that reviewer excluded. Reviewer expertise was described but not formally tested, as only one reviewer held attending status. A two-sided alpha of 0.05 was used throughout, and full model estimates are reported in Tables 1, 2, 3 and 4.

**Table 1:** Detection accuracy: descriptive and mixed-effects model results

Comparison	*n*	% Correct	OR	95% CI	*p*
*Level A: human vs. AI (collapsed)*					
Human (reference)	117	62.4	–	–	–
AI (all models)	349	49.3	0.56	0.36–0.88	0.011
*Level B: individual source*					
Human (reference)	117	62.4	–	–	–
Claude Sonnet 4.6	115	40.0	0.37	0.22–0.65	< 0.001
GPT-5.5	118	56.8	0.78	0.46–1.33	0.360
Gemini 3 Flash	116	50.9	0.60	0.35–1.03	0.065

Omnibus likelihood ratio test (source): χ²(3) = 13.86, *p* = .003

Estimates are odds ratios (OR) with 95% confidence intervals from generalized linear mixed models (binomial family, logit link) with crossed random intercepts for reviewer and abstract. The Human (reference) rows serve only as the reference category; their OR and 95% CI cells are therefore shown as dashes, and no p value is reported. Remaining rows are odds ratios relative to the human reference, so values below 1 indicate lower odds of correct classification than for human-written abstracts. “Unsure” judgments (*n* = 14) were excluded

### Ethics statement

This study involved the evaluation of previously published, de-identified abstracts by clinician reviewers. No patient data or identifiable personal information was accessed, and reviewers provided evaluations in a professional capacity without collection of personal or sensitive data. Institutional review board approval was therefore not required.

## Results

Across all 480 reviewer-abstract judgments, 14 (2.9%) were classified as “Unsure” and excluded from accuracy analyses, leaving 466 classifiable judgments. Overall classification accuracy for AI-generated abstracts, collapsed across all three LLMs, was 49.3% (*n* = 349), approximately 50%, whereas accuracy for human-written abstracts was 62.4% (*n* = 117; Table 1). The effect of true authorship on classification accuracy was significant in the mixed-effects model (*p* = .011).

**Fig. 1:**
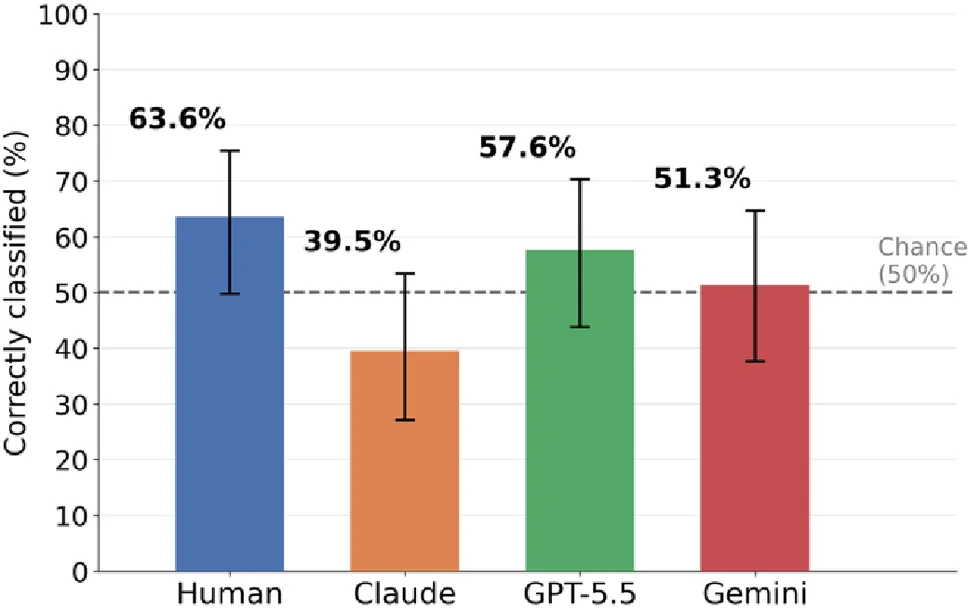
Detection accuracy by abstract source. Bars show model-estimated probabilities of correct classification derived from the Level B mixed-effects model (Table 1), with 95% confidence intervals accounting for the repeated-measures structure of the data. “Unsure” responses were excluded. The dashed line indicates chance-level performance (50%). Claude-generated abstracts were correctly identified significantly less often than human-written abstracts

When the four sources were considered individually, the omnibus likelihood ratio test indicated a significant overall effect of source on classification accuracy, χ²(3) = 13.86, *p* = .003 (Table 1; Fig. 1). Reviewers correctly identified Claude-generated abstracts as AI-written only 40.0% of the time, significantly less often than human-written abstracts were correctly identified (*p* = .003, Holm-corrected; Table 2). GPT-generated abstracts (56.8%) and Gemini-generated abstracts (50.9%) did not differ significantly from human abstracts in detectability (*p* = .692 and *p* = .261, respectively). Claude-generated abstracts were also significantly harder to detect than GPT-generated abstracts (*p* = .038); no other pairwise contrast reached significance after Holm correction.

**Table 2:** Pairwise comparisons of detection accuracy between sources

Contrast	OR	95% CI	*p* (Holm)
Human/Claude	2.67	1.28–5.57	0.003
Human/GPT-5.5	1.29	0.62–2.64	0.692
Human/Gemini	1.66	0.80–3.42	0.261
Claude/GPT-5.5	0.48	0.23–0.99	0.038
Claude/Gemini	0.62	0.30–1.28	0.261
GPT-5.5/Gemini	1.29	0.63–2.64	0.692

Odds ratios and 95% confidence intervals derived from the Level B mixed-effects model in Table 1; p values Holm-corrected for six comparisons, and confidence intervals Bonferroni-adjusted for six estimates. Values above 1 indicate higher odds of correct classification for the first-named source

**Fig. 2:**
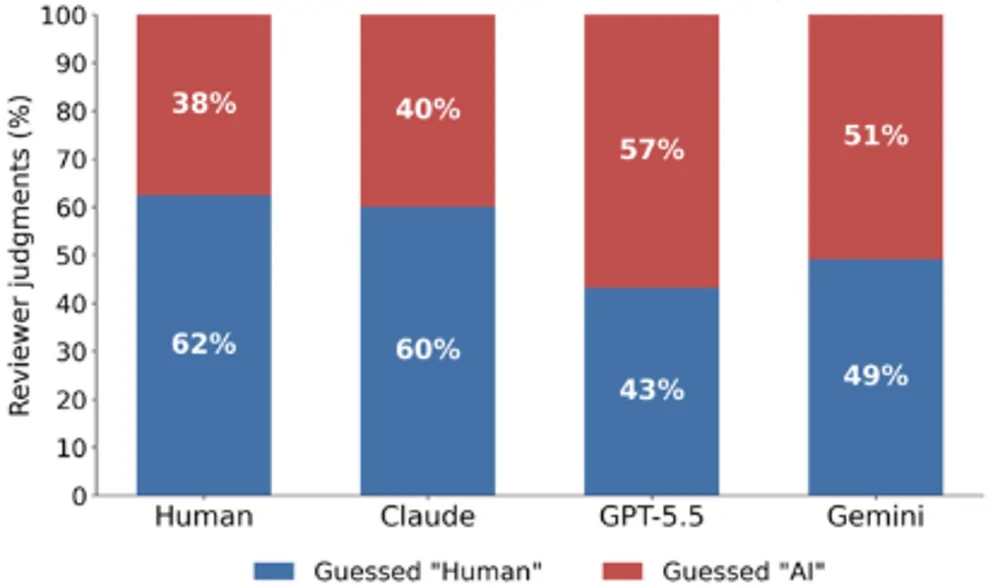
Reviewer judgment distribution by true abstract source. Stacked bars show the percentage of classifiable judgments recorded as “Human” or “AI” for each source. “Unsure” responses (*n* = 14, 2.9%) are excluded, so denominators match those used in the accuracy analyses reported in the text; their distribution by source is given in Table 4, panel B. Claude-generated abstracts were judged “Human” at a rate closely approaching that of genuinely human-written abstracts

Considering the distribution of judgments by source (Fig. 2), Claude-generated abstracts were called “Human” in 60.0% of classifiable judgments, close to the 62.4% rate for genuinely human-written abstracts. GPT and Gemini abstracts were called “Human” far less often, in 43.2% and 49.1% of judgments, respectively.

Recoding “Unsure” responses as incorrect classifications rather than excluding them did not alter any substantive conclusion (Table 4, panel D). Under this coding, accuracy was 60.8% for human-written abstracts, 38.3% for Claude, 55.8% for GPT-5.5, and 49.2% for Gemini. The effect of true authorship remained significant (*p* = .010), as did the omnibus effect of source, χ²(3) = 14.78, *p* = .002. Claude-generated abstracts remained significantly harder to detect than human-written abstracts (*p* = .002, Holm-corrected) and than GPT-generated abstracts (*p* = .024), while GPT and Gemini again did not differ significantly from human abstracts (*p* = .560 and *p* = .235). All p values were marginally smaller under this coding than in the primary analysis.

Because the four abstracts derived from each source study constitute a matched set, both accuracy models were refitted with source study added as a third random intercept. The estimated study-level variance was effectively zero, the addition did not improve model fit (likelihood ratio test, χ²(1) = 0.00, *p* = 1.00, for both models), and all fixed-effect estimates were unchanged to three decimal places. An equivalent model in which source study replaced abstract as the second random intercept produced identical estimates. Clustering by source study therefore had no detectable influence on the results.

**Fig. 3:**
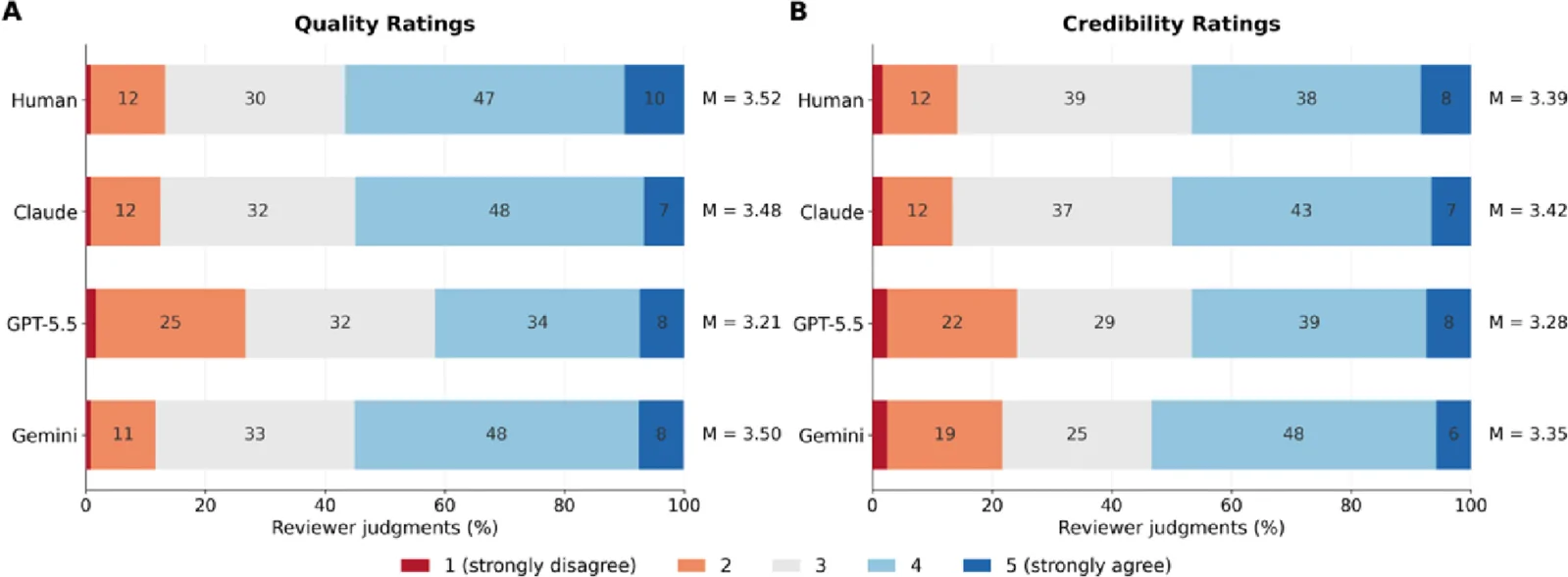
Perceived quality (**A**) and credibility (**B**) ratings by abstract source. Stacked bars show the percentage distribution of all 5-point ratings for each source (*n* = 120 judgments per source). Segments representing under 4% of judgments are drawn but not labelled. Group means are shown at right. Quality ratings differed significantly by source (χ²(3) = 8.98, *p* = .030), driven by lower ratings for GPT-5.5 (*p* = .046 vs. human, Holm-corrected); credibility ratings did not differ by source (χ²(3) = 1.98, *p* = .577)

Ratings of perceived quality differed significantly by source, χ²(3) = 8.98, *p* = .030 (Table 3; Fig. 3). Abstracts generated by GPT received significantly lower quality ratings than human-written abstracts (M = 3.21 vs. 3.52; *p* = .046, Holm-corrected), while Claude-generated (M = 3.48) and Gemini-generated abstracts (M = 3.50) did not differ significantly from human abstracts. Ratings of perceived credibility did not differ significantly by source, χ²(3) = 1.98, *p* = .577 (Fig. 3B), and no pairwise contrasts were computed for this outcome.

**Table 3:** Quality and credibility ratings by abstract source

Source	*n*	Quality, M (Mdn)	Credibility, M (Mdn)
Human	120	3.52 (4)	3.39 (3)
Claude Sonnet 4.6	120	3.48 (4)	3.42 (3.5)
GPT-5.5	120	3.21 (3)	3.28 (3)
Gemini 3 flash	120	3.50 (4)	3.35 (4)

Cumulative link mixed model results (omnibus test)			
Outcome	χ²(3)	p	Significant Holm-corrected contrast
Quality	8.98	0.030	Human − GPT-5.5 (*p* = .046)
Credibility	1.98	0.577	None (omnibus n.s.)

Cumulative link mixed models included crossed random intercepts for reviewer and abstract; Holm correction was applied only where the omnibus test was significant

*M* mean, *Mdn* median, on a 5-point scale (1 = strongly disagree, 5 = strongly agree)

**Table 4:** Inter-rater reliability, “Unsure” judgments, and sensitivity analyses

A. Inter-rater reliability		
Measure	Value	p
Fleiss’ κ (Human/AI/Unsure judgment)	− 0.009	0.674
ICC(2,k) — Quality	0.250	0.014
ICC(2,k) — Credibility	0.057	0.340

B. “Unsure” judgments by source		
Source	n	Unsure, n (%)
Human	120	3 (2.5%)
Claude Sonnet 4.6	120	5 (4.2%)
GPT-5.5	120	2 (1.7%)
Gemini 3 Flash	120	4 (3.3%)
Fisher’s exact test (Unsure × source)	—	*p* = .802

C. Sensitivity analysis (one reviewer excluded)		
Comparison	Full sample (p)	Excl. reviewer (p)
Human vs. AI (collapsed)	0.011	< 0.001
Human vs. Claude	< 0.001	< 0.001
Human vs. GPT-5.5	0.360	0.039
Human vs. Gemini	0.065	0.004

D. Sensitivity analysis (“Unsure” coded as incorrect)		
Source	% Correct, primary	% Correct, “Unsure” incorrect
Human	62.4	60.8
Claude Sonnet 4.6	40.0	38.3
GPT-5.5	56.8	55.8
Gemini 3 Flash	50.9	49.2

Comparison	Primary (*p*)	“Unsure” incorrect (*p*)
Omnibus test of source	0.003	0.002
Human vs. AI (collapsed)	0.011	0.010
Human/Claude	0.003	0.002
Human/GPT-5.5	0.692	0.560
Human/Gemini	0.261	0.235
Claude/GPT-5.5	0.038	0.024

ICC(2,k) = intraclass correlation coefficient, two-way random-effects, average-measures. In panel C, the Level A and Level B mixed-effects models (Table 1) were re-fit with one reviewer, responsible for 9 of the 14 “Unsure” responses, excluded; p values are uncorrected model contrasts against the human reference, as in Table 1, and are therefore not directly comparable with the Holm-corrected pairwise values in Table 2. In panel D, “Unsure” judgments were retained and coded as incorrect rather than excluded, so percentages are of all 120 judgments per source, whereas primary percentages are of classifiable judgments only (*n* = 115–118 per source). Pairwise p values in panel D are Holm-corrected as in Table 2; omnibus and Human vs. AI values are likelihood ratio and model contrast tests as in Table 1

Abstract characteristics by source are reported in Supplementary Table S1. Mean length did not differ significantly between sources (human 315.9 words; Claude 282.5; GPT-5.5 290.0; Gemini 264.8; F(3,42) = 2.15, *p* = .109), but variability in length did: human abstracts ranged from 135 to 572 words (SD 98.5), compared with 241 to 377 words across all model-generated abstracts (SD 32.5; Fligner–Killeen χ²(3) = 10.93, *p* = .012). The number of reported numerical values differed markedly by source, F(3,42) = 23.19, *p* < .001, with GPT-5.5 abstracts containing approximately twice as many as those from any other source (M = 65.8, vs. 29.9 for human, 36.3 for Claude, and 34.5 for Gemini).

**Fig. 4:**
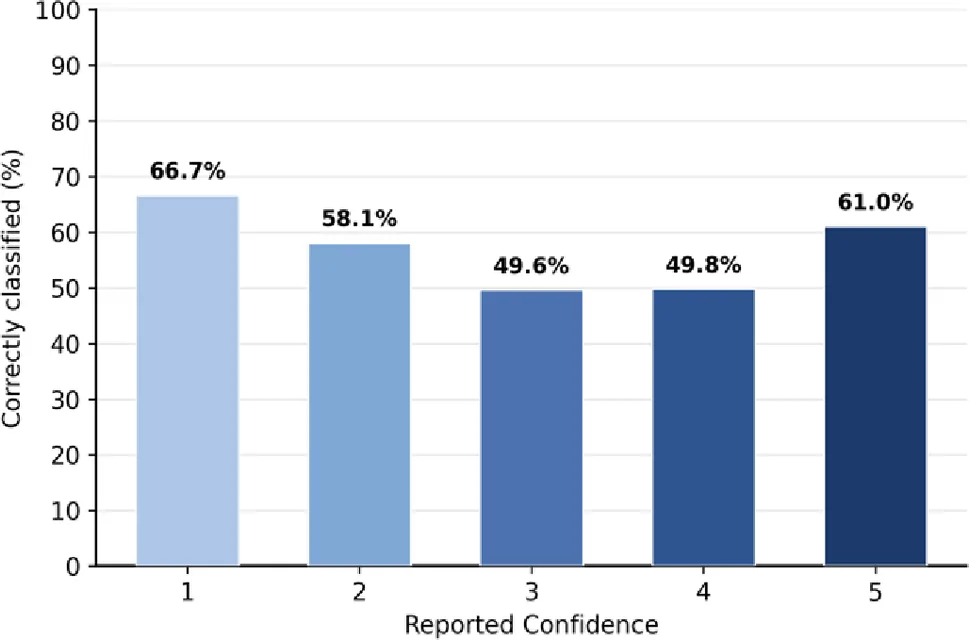
Classification accuracy by reviewer-reported confidence. Confidence was rated on a 5-point scale (1 = not confident, 5 = extremely confident). Sample sizes per confidence level (1–5) were *n* = 9, 62, 133, 203, and 59. Chance-level performance = 50%. Accuracy did not increase monotonically with confidence, and confidence did not predict correct classification (*p* = .749)

Reviewers’ self-reported confidence did not predict the accuracy of their classifications (*p* = .749; Fig. 4). Accuracy did not increase monotonically with confidence: the highest accuracy was observed at the lowest and highest confidence levels (66.7% at a confidence of 1, *n* = 9; 61.0% at a confidence of 5, *n* = 59), with accuracy at intermediate confidence levels near chance (58.1%, 49.6%, and 49.8% at confidence levels 2, 3, and 4; *n* = 62, 133, and 203, respectively).

Inter-rater agreement on the categorical Human/AI/Unsure judgment was negligible (Fleiss’ κ = −0.009, *p* = .674). Agreement on quality ratings was low (ICC(2,k) = 0.250, *p* = .014), and agreement on credibility ratings was not significant (ICC(2,k) = 0.057, *p* = .340; Table 4).

“Unsure” judgments did not differ significantly by abstract source (Fisher’s exact test, *p* = .802); across sources, the frequency of “Unsure” responses ranged from 1.7% to 4.2% (Table 4). One reviewer accounted for 9 of the 14 total “Unsure” responses (15.0% of that reviewer’s judgments), compared with 0%–6.7% for the remaining seven reviewers. With this reviewer excluded, the effect of source on detection accuracy remained significant for Claude (*p* < .001) and became significant for both GPT (*p* = .039) and Gemini (*p* = .004), which had not reached significance in the full sample.

Because only one of the eight reviewers held attending-level status, expertise could not be statistically disentangled from individual reviewer identity and was examined descriptively only. Raw classification accuracy was similar between the attending reviewer (54.9%) and the seven resident reviewers (52.3%).

Qualitative review of free-text justifications indicated that reviewers relied principally on impressions of genericness and on the absence of concrete clinical detail when AI authorship was suspected, with stylistic naturalness and the specificity of reported findings cited most often as the basis for judgment.

## Discussion

The principal finding of this study is that urologists had only a limited ability to distinguish AI-generated from human-written abstracts describing the same underlying studies. Overall AI-authorship detection accuracy was approximately 50%, and human-written abstracts were classified correctly significantly more often, a pattern driven disproportionately by one model.

Overall accuracy of 49.3% places this study toward the harder end of a mixed literature. Gao et al. reported 68% accuracy for GPT-generated biomedical abstracts, better than the present result, although those reviewers also described suspected AI text as vague and formulaic [6]. Hakam et al. found orthopedic reviewers and AI-detection software alike unable to reliably identify AI-generated texts [11]. Silva et al., in a blinded essay contest for the journal Stroke, found accuracy of exactly 50%, described by the authors as essentially a coin flip and the closest direct analogue to the present result [8]. 

A contemporaneous otolaryngology study by Hack et al. used a nearly identical design, with twenty clinician reviewers and four LLMs including Claude and GPT variants, and found similarly low overall accuracy (44.7%) [13]. Notably, the direction of the human–AI asymmetry was reversed in that study, with human abstracts more often misclassified as AI [13]. This is a useful contrast rather than a contradiction. It suggests that which category “looks more human” may depend on specialty conventions and on the specific comparison texts and models used, rather than reflecting a fixed property of AI writing in general.

Detectability was significantly model-specific in the present data. Claude was correctly identified as AI only 40.0% of the time, significantly less often than human abstracts and less often than GPT, while GPT and Gemini did not differ significantly from the human baseline. This is corroborated directly by Hack et al., who also found Claude among the models most often mistaken for human, while a different model, Grok, was by far the easiest to detect [13]. Two independent studies in different specialties reaching the same conclusion about one model specifically is stronger evidence than either result alone, and argues against treating “AI-generated text” as a single uniform category. Only the Claude comparison was stable across sensitivity analyses. Excluding the single reviewer responsible for most “Unsure” responses left the Claude finding essentially unchanged but moved both the GPT and Gemini comparisons from non-significant to significant (*p* = .039 and *p* = .004, respectively). That a single reviewer of eight can determine whether two of three model comparisons reach significance is itself an indication of how unstable these estimates are at this sample size, and the GPT and Gemini results should accordingly be regarded as exploratory rather than confirmed. The model-specific pattern was also unaffected by clustering at the level of the source study, which is expected given that every study contributed exactly one abstract per source, so study-level variation shifts all four conditions equally and cancels in the between-source contrasts.

The finding that 60% of Claude abstracts were judged “Human,” nearly matching the 62.4% rate for genuinely human abstracts, is the clearest illustration of this effect and the headline practical result of the study. It is not simply a general bias toward guessing “human,” since such a bias would apply evenly across all AI sources rather than concentrating on one model. This fits the report of Kresoja et al., who found no significant difference in blinded ratings between AI-generated and human-authored abstracts submitted to a real cardiology congress, supporting functional indistinguishability as a realistic possibility in actual review workflows [14]. 

Detectability and perceived quality were separable constructs. GPT was rated significantly lower in quality than human abstracts, while Claude and Gemini did not differ from human writing on this measure, and credibility ratings showed no source effect at all. GPT was therefore both the easiest to detect and the lowest rated, whereas Claude was the hardest to detect yet rated no differently from human work. Hack et al. reported a comparable quality gap in the opposite comparison, with human abstracts modestly outscoring AI overall, and Silva et al. found AI essays rated higher for composition quality despite chance-level detection, alongside a strong bias against essays once suspected of being AI-written [8, 13]. Together these observations suggest that quality perception may be adjusted after authorship is suspected, rather than driving that suspicion. Information density offers one candidate explanation for GPT’s position at both extremes. GPT’s abstracts contained roughly twice as many numerical values as those from any other source, and dense enumeration of figures may have read as mechanical, contributing both to lower perceived quality and to more frequent identification as machine-written.

Reviewer confidence did not predict accuracy in this study, and the raw pattern was not even monotonic. Hack et al. reported the identical disconnect, describing reviewers as sometimes highly confident in incorrect classifications [13]. Liang et al. found a related pattern in AI-conference peer review, where reviews estimated to contain substantial LLM-generated text were associated with lower reviewer confidence [19]. Confidence should not be treated as a proxy for reliability in any workflow that relies on human judgment to flag AI content.

Inter-rater agreement was negligible, indicating that reviewers were not simply inaccurate but also not converging on the same texts. Hakam et al. similarly found no relationship between reviewer seniority or specialty and detection ability [11]. Free-text rationales in the present study centered on genericness and lack of clinical detail, closely matching the themes Hack et al. reported of vagueness, odd phrasing, and absence of data [13]. This convergence suggests that these are the general heuristics clinicians currently rely on, and that both studies found them only weakly predictive of actual accuracy.

Taken together, these findings have direct implications for editorial practice. If detectability is model-specific and shifting, then policies framed around the assumption that AI writing has a recognizable signature are unlikely to remain durable. The combination of approximately 50% overall AI-authorship detection accuracy, uncalibrated confidence, and negligible agreement observed here suggests that unaided human review functions poorly as a screening mechanism, and that disclosure requirements and hybrid human–algorithmic approaches are the more defensible safeguards.

### Limitations

This study has several limitations. The reviewer sample was small, and expertise could not be separated from individual reviewer identity, since only one reviewer held attending status. Negligible inter-rater agreement indicates that the measured detectability likely depends partly on which reviewers were sampled. All reviewers saw the abstracts in the same order, so order effects could not be separated from abstract identity. Source studies were selected by convenience rather than by a formal random or consecutive procedure, and the set comprised only 15 studies; although these spanned several urological subspecialties and study designs, detectability may depend on the genre of the source study, and a differently weighted set could yield different stylistic cues. The task involved abstracts rather than full manuscripts, which narrows the cues available and may partly explain the low accuracy relative to some full-text studies. Relatedly, because the models generated new abstracts rather than rewriting the published one, the comparison captures writing style together with content selection and organisation; a design in which models rewrite the original abstract while preserving its content would isolate linguistic style more strictly, at the cost of realism.

Although mean abstract length did not differ significantly between sources, human abstracts varied far more in length than model-generated ones, which were constrained by the prompt: the three shortest and the single longest abstract were all human, as were the three containing almost no numerical results. Unusually short, long, or non-quantitative abstracts may therefore have offered an incidental cue to human authorship. As in Hack et al., human comparison abstracts drawn from the current publishing era cannot be guaranteed free of undisclosed AI-assisted editing.¹³ Generation used default consumer interfaces, whose memory, routing, and tool behaviours are neither visible to nor controllable by the investigator and change over time; conditions are documented as fully as the interfaces permit but cannot be exactly reproduced. Finally, because model performance differed substantially by source, these results should not be generalized to other LLMs or to future versions of the same models.

### Future directions

Larger reviewer panels, including journal editors and biostatisticians, would allow expertise to be modeled as an independent factor rather than reported as a descriptive note. Extending this design to full manuscripts, rather than abstracts alone, would clarify whether additional textual cues improve detection. Repeating this design as models are updated would help establish whether detectability is stable or continuing to shift, particularly for models that already approach human-level indistinguishability. Combining human review with technical detection tools, rather than relying on either alone, is a natural next step given the poor performance and poor agreement of unaided human judgment observed here and elsewhere.

## Conclusions

Reviewers in this study could not reliably distinguish AI-generated from human-written urology abstracts, and this difficulty was concentrated in one model, Claude, whose abstracts were judged nearly as human as genuine human writing. Perceived quality and credibility did not track actual detectability, and neither reviewer confidence nor inter-rater agreement provided any protective signal. These findings, corroborated by an independent and closely comparable study in otolaryngology, indicate that unaided human review is not a dependable safeguard against undisclosed AI authorship, at least at the abstract level, and that editorial policy should be built around the expectation that detectability will continue to shift as individual models improve, rather than around any fixed assumption about what AI-written text looks like.

## Supplementary Information

Below is the link to the electronic supplementary material.

Supplementary Material 1

Supplementary Material 2

## Data Availability

No datasets were generated or analysed during the current study.
