# Supplementary material for: Confidence without consensus? Human versus AI abstracts in urology
Source: World J Urol. 2026 Oct 16;44(1):751. doi: 10.1007/s00345-026-06872-6 (PMC13675285; doi:10.1007/s00345-026-06872-6)
Supplement: Supplementary file 1 [file 345_2026_6872_MOESM1_ESM.docx]

***ABSTRACT 1***

Human

Word count: 461

**Background:** Treatments for men seeking fertility care are limited. Antioxidant supplements have been widely studied as a new treatment option, but these studies have had conflicting results. To assess whether treatment of men seeking fertility care with an antioxidant supplement can improve semen quality and pregnancy rates compared with a placebo.

**Methods:** The SUMMER trial was a multicenter, double-blind, placebo-controlled randomized clinical trial conducted in 21 hospitals and private fertility clinics in the Netherlands. Male patients in these centers were enrolled between May 2018 and February 2024, and follow-up of the primary outcome was completed in December 2024. Eligible participants were men aged 18 to 50 years with female partners aged 18 to 43 years, who sought fertility care and were advised to undergo expectant management, treatment with intrauterine insemination, in vitro fertilization (IVF), or intracytoplasmic sperm injection (ICSI). Couples treated with ovulation induction only or IVF for bilateral tubal pathology were excluded. The men were randomly assigned to receive an antioxidant supplement or a placebo. Intention-to-treat analysis was performed for all outcomes. The antioxidant supplement (Impryl) was a tablet to be taken daily for 6 months. It contained betaine (200 mg), L-cystine (200 mg), niacin (16 mg), zinc (10 mg), vitamin B6 (1.4 mg), vitamin B2 (1.4 mg), folic acid (400 µg), and vitamin B12 (2.5 µg). The placebo tablet and its packaging were identical to those of the antioxidant supplement. All participating couples received standard infertility care. The primary outcome was ongoing pregnancy conceived within 6 months after randomization. Secondary outcomes included semen parameters, sperm DNA fragmentation, fertilization and embryo utilization rates after IVF or ICSI, biochemical and clinical pregnancy rates, first-trimester pregnancy loss, ectopic pregnancy rate, cumulative number of pregnancies, time to pregnancy, and adverse events.

**Results:** A total of 1171 men (median [IQR] age, 34 [31-38] years; female partners' median [IQR] age, 32 [30-35] years) were included in the data analysis, of whom 591 were in the antioxidant supplement group and 580 were in the placebo group. Ongoing pregnancy rate within 6 months was not significantly different between the 2 groups (193 of 571 [33.8%] vs 208 of 555 [37.5%]; adjusted odds ratio [AOR], 0.85 [95% CI, 0.66-1.09]; P = .20). Within the window of optimal treatment effect between 4 and 6 months (considering a spermatogenesis cycle of 72 days), ongoing pregnancy rate was significantly lower in the antioxidant supplement group compared with the placebo group (69 of 446 [15.5%] vs 95 of 442 [21.5%]; AOR, 0.66 [95% CI, 0.47-0.94]; P = .02). There were no significant between-group differences for the secondary outcomes.

**Conclusions:** This randomized clinical trial found that ongoing pregnancy rates did not improve with the antioxidant supplement compared with a placebo. Therefore, the investigators do not support its use in men seeking fertility care.

***Abstract 2***

Gemini

Word count: 251

**Background**: The objective of this study was to conduct an in-depth, large-scale, real-world analysis of post-Aquablation bleeding outcomes and assess the global utilization of this robotically executed waterjet tissue resection technique for the treatment of bladder outlet obstruction due to benign prostatic hyperplasia.

**Methods**: This study utilized a prospective case-detail database merged with the United States Food and Drug Administration Manufacturer and User Facility Device Experience (MAUDE) database. Researchers analyzed procedural data from 2019 to 2024 across Asia, Europe, and North America. Outcomes were evaluated based on prostate size distribution and the incidence of bleeding-related complications, specifically defined as blood transfusion or surgical takeback for haemostatic cautery.

**Results**: Analysis of 70,270 procedures revealed a mean prostate volume of 87.3 (SD 42.4) mL, with a maximum volume of 1189 mL. North America reported the highest proportion of large prostates, with 46.1% measuring >80 mL. The overall pooled rate for both transfusion and takeback for cautery was 0.2%. Annual transfusion rates decreased from 2.3% in 2019 to 0.1% in 2023 and 2024. Surgical takeback rates for haemostatic cautery declined from 2.7% in 2019 to 0.1% between 2022 and 2024. Compared to transurethral resection of the prostate, Aquablation demonstrated significantly lower odds of transfusion (odds ratio 0.60; 95% CI 0.47–0.76; P < 0.001).

**Conclusion**: Aquablation has achieved widespread global adoption across all prostate sizes, including very large glands. The consistent reduction in transfusion and surgical takeback rates over time highlights significant advancements in the safety profile and perioperative haemostatic management of the procedure.

***Abstract 3***

GPT5

Word count: 261

**Background:** To compare prone versus supine positioning during suction mini-percutaneous nephrolithotripsy for stone-free status and complications.

**Methods:** Adults with kidney stones enrolled between March and November 2024 across 30 centers in 21 countries were analyzed. Suction mini-PCNL used a 14–22 Fr suction sheath. Thirty-day computed tomography defined grade A as zero residual fragments, grade B as a single residual fragment ≤4 mm, and grade C as residual fragments >4 mm or multiple residual fragments. Multivariable models evaluated grade A status and complications.

**Results:** Among 1534 patients, 653 (43%) underwent prone and 881 (57%) supine surgery. Median age was 49 years (IQR 39–59) versus 50 years (IQR 39–62; p=0.14), body mass index 25.0 kg/m² (IQR 22.3–28.0) versus 27.2 kg/m² (IQR 24.2–30.6; p<0.001), stone density 1159 (IQR 898–1300) versus 1200 Hounsfield units (IQR 950–1400; p<0.001), and largest stone diameter 1.8 cm (IQR 1.5–2.4) versus 2.1 cm (IQR 1.6–2.8; p<0.001). Blood transfusion occurred in 18 (2.8%) versus 0 (0%; p<0.001), renal pelvic perforation in 18 (2.8%) versus 2 (0.23%; p<0.001), and pneumothorax in 10 (1.5%) versus 0 (0%; p<0.001). Grade A status was 555 (85%) versus 717 (81%; p=0.052); readmission within 72 hours was 10 (1.5%) versus 16 (1.8%; p=0.7). Supine positioning was not associated with grade A status (adjusted odds ratio 0.92, 95% CI 0.66–1.29; p=0.6) or complications (adjusted odds ratio 0.87, 95% CI 0.59–1.28; p=0.5).

**Conclusion:** Prone and supine suction mini-PCNL showed comparable grade A stone-free rates and complication rates. Position was not independently associated with grade A status or complications.

***Abstract 4***

Gemini

Word count: 305

**Background:** The primary aim of this randomized controlled trial was to evaluate the effects of a patient-oriented online decision aid versus a standard printed brochure on treatment decisions and secondary outcomes in men diagnosed with nonmetastatic prostate cancer.

**Methods** This randomized controlled prospective study recruited male patients aged 18–80 years with histologically confirmed adenocarcinoma of the prostate across 116 urological practices in Germany. Participants were randomized 1:1 to receive either a web-based online decision aid or a 100-page printed brochure. The primary outcome was the treatment decision at 14 months, specifically aiming for guideline-conforming use of observation. Secondary outcomes, including decisional conflict, knowledge scores, and quality of life, were measured at 4-week and 14-month follow-ups using validated questionnaires.

**Results** A total of 1115 patients were randomized, and data from 1000 patients (516 intervention, 484 control) were available for final analysis. There was no significant difference in the proportion of observation between the intervention and control groups (15.3% vs 17.4%, P = 0.4). For low/early-intermediate risk patients, observation rates were 39.7% for intervention and 41.1% for control (P = 0.8). At 14 months, only satisfaction with doctor-patient communication showed a significant difference favoring the intervention group (88.4% vs 86.2%, P = 0.044). Patients in the intervention group reported significantly shorter consultation times (61.1 ± 45.2 min vs 69.4 ± 55.6 min, P = 0.02) and more frequent summary preparation (75.8% vs 56.0%, P < 0.001).

**Conclusion** The online decision aid demonstrated equivalence to the current gold-standard printed brochure in influencing primary treatment decisions for nonmetastatic prostate cancer. While it did not significantly alter the rates of guideline-compliant observation, the digital tool was effectively implemented and associated with improved counseling efficiency and patient involvement. These findings suggest that digital health applications can be integrated into routine urological care as an effective alternative to traditional information materials.

***Abstract 5***

Claude

Word count: 282

**Background:** Aquablation is an ultrasound-guided, robotically executed waterjet tissue resection technique for benign prostatic hyperplasia (BPH). While its safety and efficacy have been established in clinical trials, real-world data on post-procedural bleeding complications across diverse prostate sizes and global practice settings remain limited. This study aimed to conduct a large-scale analysis of post-Aquablation bleeding outcomes and assess utilisation trends using global real-world data from 2019 to 2024.

**Methods:** A prospective manufacturer-maintained database incorporating procedural data collected by on-site representatives was merged with adverse event data from the United States Food and Drug Administration Manufacturer and User Facility Device Experience (MAUDE) database. Patients from Asia, Europe, the Middle East, and North America who underwent Aquablation between 2019 and 2024 were included. Primary outcomes were transfusion rate and surgical takeback rate for haemostatic cautery, evaluated annually and stratified by region.

**Results:** A total of 70,270 Aquablation procedures were performed across the study period. The mean prostate volume was 87.3 (SD 42.4) mL, with a maximum recorded volume of 1,189 mL. North America had the highest proportion of large prostates, with 46.1% measuring >80 mL. Transfusion rates declined from 2.3% in 2019 to 0.1% in 2024, yielding an overall pooled rate of 0.2%. Takeback rates for cautery decreased from 2.7% in 2019 to 0.1% between 2022 and 2024, with an overall pooled rate of 0.2%. Regional transfusion rates were 0.2% in North America, Europe, the Middle East, and Africa, and 0.1% in Asia.

**Conclusion:** This large-scale real-world analysis of 70,270 Aquablation procedures demonstrates consistent adoption across all prostate sizes with a markedly favourable safety profile. Transfusion and surgical takeback rates declined substantially between 2019 and 2024, confirming significant improvements in perioperative haemostatic management over time.

***Abstract 6***

Human

Word count: 135

**Background**: Treatment of erectile dysfunction is based on pharmacotherapy for most patients. To review the current data on pharmacotherapy for erectile dysfunction based on efficacy, psychosocial outcomes, and safety outcomes.

**Methods**: A review of the literature was undertaken by the committee members. All related articles were critically analyzed and discussed, and consensus statements were developed after presentation at the 2024 ICSM.

**Results**: Eight recommendations are provided with the corresponding level of evidence and grade of recommendation.

**Conclusions**: The management of erectile dysfunction should be personalized to address the psychosocial needs and expectations of both the patient and their partner. PDE5 inhibitors remain the first-line treatment for most men, while intracavernosal injections, vacuum erection devices, and penile prostheses serve as second-line options, with treatment decisions guided by patient preferences. Key recommendations are summarized in table 1.

***Abstract 7***

Gemini

Word Count: 273

**Background:** The study aimed to compare the safety and efficacy of Aquablation and laser enucleation of the prostate (LEP) for the treatment of lower urinary tract symptoms in men with large prostate glands ranging between 80 and 180 ml.

**Methods:** WATER III was an investigator-initiated, prospective, multicenter trial utilizing a Bayesian hierarchical model to combine data from randomized and nonrandomized cohorts. Participants were men aged 45–80 years with IPSS ≥8 and prostate volumes of 80–180 ml. The primary efficacy endpoint was the change in IPSS from baseline to 3 months, with a noninferiority margin of 4.7. The primary safety endpoint was the occurrence of Clavien-Dindo grade ≥2 or persistent grade 1 adverse events.

**Results:** Of 202 participants, 98 underwent Aquablation (mean age 65.2 ± 6.8 years) and 88 underwent LEP (mean age 67.5 ± 6.6 years; p=0.02). At 3 months, mean IPSS reduction was 12.9 ± 6.9 for Aquablation and 13.1 ± 7.5 for LEP, demonstrating noninferiority (posterior probability >0.999). Safety events occurred in 40.8% of Aquablation versus 56.8% of LEP patients. Persistent de novo ejaculatory dysfunction was significantly lower after Aquablation (14.8%) than LEP (77.1%; p < 0.0001). Persistent de novo stress urinary incontinence was 0% for Aquablation and 9.3% for LEP (p < 0.05). LEP showed greater mean Qmax improvement (27.5 ± 11.3 vs 19.8 ± 10.6 ml/s; p < 0.0001) and greater prostate volume reduction (75% vs 50%; p < 0.0001).

**Conclusion:** Aquablation demonstrated noninferior short-term symptom relief and superior preservation of sexual and urinary functions compared to LEP. Although LEP achieved greater flow rate improvement and volume reduction, Aquablation is an effective, safe alternative for large prostates.

***Abstract 8***

Claude

Word count: 266

**Background:** Intravaginal electrical stimulation (IVES) is a minimally invasive treatment for urinary incontinence (UI) in women, yet its efficacy compared to other conservative interventions remains unclear. This systematic review evaluated the clinical utility of IVES against other nonsurgical interventions for UI in women by comparing outcomes including urinary leakage, patient-reported outcome measures, pelvic floor muscle (PFM) strength, and quality of life (QoL).

**Methods:** This systematic review was conducted in accordance with PRISMA guidelines and registered with PROSPERO (CRD42022318840). Systematic searches were performed across PubMed, EMBASE, Ovid Medline, and IEEE Xplore up to November 9, 2020, with updated searches on August 7, 2023, and February 7, 2025. Randomized controlled trials evaluating IVES for UI or pelvic organ prolapse in women, compared to conservative nonsurgical and nonpharmacological treatments, were included. Risk of bias was assessed using the Cochrane Risk of Bias 2.0 tool.

**Results:** From 4,898 identified records, 32 RCTs were included. UI subtypes investigated were stress (n=19), mixed (n=10), urgency (n=14), and unspecified UI (n=3). Of 15 trials measuring pad weight, 10 (66.7%) demonstrated significant within-group reductions for IVES. Six of 8 remaining trials (75%) reported significant within-group reductions in pad usage. Nineteen trials examined PFM strength, with 11 revealing statistically significant within-group improvements. Of 27 trials evaluating QoL, 15 (78.9%) showed significant within-group improvements. Adherence rates ranged from 61% to 100%. Adverse events were generally low-to-moderate in severity.

**Conclusion:** IVES may be a safe approach for UI in women, demonstrating potential benefits in pad weight reduction, PFM strength, and QoL. However, the evidence is of low quality, and adequately powered RCTs with standardized protocols are needed.

***Abstract 9***

Human

Word count: 335

**Background:** Urotherapy remains the first-line conservative treatment of pediatric bowel and bladder dysfunction (BBD); however, some patients show limited or no response. Early identification of patients likely to fail urotherapy alone could influence early management and outcomes. This study aimed to develop predictive models to identify pediatric patients unlikely to respond to urotherapy alone.

**Methods:** A retrospective cohort of 123 pediatric patients aged 5–10 years diagnosed with BBD who completed a validated 18-question BBD symptom questionnaire at their initial and follow-up visit was analyzed. Patients underwent urotherapy as the primary intervention, and symptom improvement was assessed at 6 months or less through a standardized scoring system. Machine learning models, including multivariable logistic regression and random forest classifiers, were developed to identify predictors of non-response to urotherapy. Model performance was evaluated using the area under the receiver operating characteristic curve (AUROC).

**Results:** A total of 123 patients met inclusion criteria, including 92 (75%) females, with a median age of 6 years (IQR 5–8). The median time from the initial to the next follow-up visit was 3 months (IQR 1–4). Overall, 26 patients (21%) had complete improvement, 28 (23%) had moderate improvement, 23 (19%) had minimal improvement, and 46 (38%) had no improvement. Older age (OR 1.45, 95% CI 1.09–1.98; p = 0.01) and presence of dysuria (OR 1.54, 95% CI 1.06–2.37; p = 0.03) at the initial visit were associated with an increased likelihood of response to urotherapy, whereas presence of daytime incontinence (OR 0.67, 95% CI 0.46–0.97; p = 0.04) was associated with a lower likelihood of response. The logistic regression model achieved an AUROC of 0.67, while the random forest model slightly outperformed it with an AUROC of 0.71.

**Conclusion:** Machine learning models using demographic and standardized questionnaire data predicted non-response to urotherapy in pediatric patients with BBD. Age, dysuria, and daytime incontinence were identified as significant predictors. Early identification of potential non-responders could allow clinicians to implement additional therapeutic strategies sooner, potentially improving patient care and outcomes.

***Abstract 10***

Claude

Word count: 316

**Background:** To evaluate whether treatment with a combined antioxidant supplement improves semen quality and pregnancy rates in men seeking fertility care compared with placebo.

**Methods:** The SUMMER trial was an investigator-initiated, double-blind, placebo-controlled randomized clinical trial conducted from May 2018 to February 2024 across 21 centers in the Netherlands. Men aged 18 to 50 years with female partners aged 18 to 43 years, unable to conceive after 12 months of unprotected intercourse, were randomized 1:1 to receive one daily tablet of an antioxidant supplement (Impryl) or placebo for 6 months. The supplement contained betaine (200 mg), L-cystine (200 mg), niacin (16 mg), zinc (10 mg), vitamin B6 (1.4 mg), vitamin B9 (400 µg), and vitamin B12 (2.5 µg). The primary outcome was ongoing pregnancy within 6 months after randomization, defined as a viable pregnancy at 12 weeks of gestation confirmed by ultrasonography.

**Results:** Of 1217 randomized patients, 1171 were included in the primary analysis (antioxidant supplement group n = 571; placebo group n = 555). Ongoing pregnancy within 6 months did not differ significantly between groups (33.8% vs 37.5%; adjusted OR, 0.85 [95% CI, 0.66–1.09]; P = .20). Ongoing pregnancy rate within the 4-to-6-month optimal treatment window was significantly lower in the antioxidant group (15.5% vs 21.5%; AOR, 0.66 [95% CI, 0.47–0.94]; P = .02). Among IVF or ICSI patients, pregnancy rates after fresh embryo transfer (42.9% vs 56.2%; AOR, 0.53 [95% CI, 0.33–0.86]; P = .009) and after ovum pickup (57.8% vs 68.6%; AOR, 0.58 [95% CI, 0.36–0.94]; P = .03) were significantly lower in the antioxidant group. No significant differences in semen parameters were observed between groups.

**Conclusion:** Combined antioxidant supplementation did not improve ongoing pregnancy rates in men seeking fertility care and was associated with significantly lower pregnancy rates during the optimal treatment window and following IVF or ICSI procedures, providing no support for its use in this population.

***Abstract 11***

Gemini

Word count: 313

**Background** The objective of this study was to identify various types of male sexual dysfunction (MSD) associated with infertility and provide evidence-based recommendations for the clinical management of these conditions. The research aimed to address the frequent co-existence of sexual disorders and subfertility, providing a structured framework for evaluating and treating affected couples to optimize both reproductive and sexual health outcomes.

**Methods** An initial exploratory analysis identified subtopics pertaining to MSD in association with infertility. Multiple literature searches were performed using Medline and PubMed databases through June 2024 to identify pertinent studies. Summaries of available evidence were reviewed to frame provisional recommendations, which were subjected to multiple rounds of revision using a modified Delphi process until a consensus was reached. Recommendations were then graded as weak or strong using the GRADE criteria.

**Results** Infertility affects 10% of couples and 12% of men; MSD prevalence in infertile men ranges from 6.7% to 75%. Meta-analysis of 2276 infertile men compared to 1492 fertile men showed a significantly higher likelihood of erectile dysfunction (OR 2.92, 95% CI 1.71, 4.96), with rates between 17.8% and 85.9%. Tadalafil 20 mg daily demonstrated noninferiority to placebo regarding 50% or greater reduction in sperm concentration. In unconsummated marriage, a multidisciplinary approach helped 97% of 135 patients achieve penetrative intercourse. For retrograde ejaculation, combination therapy with imipramine and pseudoephedrine was successful in 61% of participants. Exogenous testosterone resulted in azoospermia in 77% of men with prior documented fertility in a median time of 100 days.

**Conclusion** Sexual dysfunction and infertility frequently co-exist as either cause or effect. Management should include detailed sexual histories and physical examinations. Evidence supports the use of phosphodiesterase 5 inhibitors, multidisciplinary care for unconsummated marriages, and specific pharmacotherapy for ejaculatory disorders. Exogenous testosterone should be avoided in men seeking fertility. Optimizing comorbid conditions and lifestyle factors, such as smoking cessation, may improve overall sexual and reproductive outcomes.

***Abstract 12***

GPT5

Word count: 377

**Background:** To assess the safety and feasibility of hydrogel-assisted stone extraction using mediNiK® during flexible ureterorenoscopy (fURS) compared with standard fURS alone in patients with kidney stones larger than 8 mm on preoperative computed tomography.

**Methods:** This single-blinded prospective randomized multicenter study was conducted between September 2021 and September 2022 at five German hospitals. Eligible patients were scheduled for elective fURS with lithotripsy for kidney stones >8 mm. Patients were randomized electronically to fURS plus mediNiK® or fURS alone. Safety was evaluated over 6 postoperative weeks using treatment-emergent adverse events (TEAEs). Feasibility outcomes included extracted fragment counts by size category, number of fragments extracted per patient, procedural duration, and surgeon-rated procedural difficulty on a 5-point Likert scale.

**Results:** Seventy patients were screened, 5 were excluded, and 65 were randomized; the safety evaluation set included 29 patients per group and the full analysis set included 23 fURS+MN and 17 fURS patients. Mean age was 52.3±15.9 versus 51.7±16.5 years, BMI was 27.6±4.44 versus 28.6±6.74 kg/m², and females comprised 10/29 (34.5%) in both groups. No severe TEAEs occurred. At least one TEAE occurred in 17.2% (5/29) versus 24.1% (7/29); procedural pain in 10.3% (3) versus 6.9% (2), urinary tract obstruction in 3.4% (1) versus 3.4% (1), urinary tract infection in 0 versus 6.9% (2), haematuria in 0 versus 3.4% (1), urinary tract pain in 0 versus 3.4% (1), and non-urinary-tract events in 10.3% (3) versus 6.9% (2). Fragments <0.5 mm numbered 1492 versus 194, 0.5 to <1 mm 224 versus 15, ≥1 mm 310 versus 118, and total fragments 2026 versus 327; fragments <1 mm totaled 1716 versus 209. Median fragments extracted per patient were 7 (21) versus 0 (2) (p=0.013). Median procedural time was 80 (28) versus 62 (20) minutes (p=0.02). Difficulty ratings were very easy 13% (3) versus 0, easy 13% (3) versus 29.4% (5), average 34.8% (8) versus 11.8% (2), somewhat difficult 26.1% (6) versus 47.1% (8), and very difficult 13% (3) versus 11.8% (2) (p=0.2942).

**Conclusion:** Hydrogel-assisted fURS with mediNiK® was feasible and showed no severe TEAEs in this cohort. Compared with fURS alone, it yielded higher fragment extraction counts across all reported size categories and a higher median number of fragments extracted per patient, but required longer procedural time, while surgeon-rated difficulty was not different.

***Abstract 13***

Gemini

Word count: 257

**Background:** This multicenter prospective randomized control trial aimed to evaluate the oncological and functional outcomes of patients undergoing artificial intelligence-driven three-dimensional augmented reality robot-assisted radical prostatectomy (3D-AR-RARP) compared to standard two-dimensional magnetic resonance imaging-guided (2D-MRI) intervention. The primary endpoint was the positive surgical margin rate (PSMr) after surgery.

**Methods:** Adult patients (≥40 years) with localized prostate cancer and suspicious extracapsular extension (ECE) or bulging on preoperative MRI were randomized in a 1:2 ratio. Interventions were performed by two surgeons. In the 3D-AR group, virtual models were overlaid onto the surgical field using convolutional neural networks, facilitating targeted excisional biopsies of neurovascular bundles (NVBs). Outcomes included PSMr, urinary continence, sexual potency, and biochemical recurrence (BCR).

**Results:** Of 138 patients assessed, 133 received the intervention (49 3D-AR; 84 2D-MRI). Preoperative characteristics were comparable; 63% and 55% had PIRADS 5 scores, and 45% and 32% had ECE in the 3D-AR and 2D-MRI groups, respectively. Operative times were 135 and 127 minutes (p=0.08). PSMr on the prostate surface was 47% (23/49) for 3D-AR versus 45% (38/84) for 2D-MRI (p=0.8). However, residual PSMs at the level of preserved NVBs after excisional biopsy were significantly lower in the 3D-AR group at 22% (11/49) versus 39% (33/84) for 2D-MRI (p=0.047). Social continence at 12 months was 94% versus 75% (p=0.03). BCR occurred in 18% (6/34) and 15% (8/53) of patients (p=0.7).

**Conclusion:** AI-driven 3D-AR technology enables precise identification of tumor lesions at the level of preserved NVBs. This guidance facilitates selective biopsies, significantly reducing the occurrence of PSMs and improving 12-month urinary continence recovery.

***Abstract 14***

Gemini

Word count: 289

**Background:** The primary objective of this study was to provide updated evidence-based standards for the management of various pediatric urological conditions, specifically addressing undescended testes (UDT), testicular tumors, acute scrotum, hypospadias, congenital penile curvature (CPC), urinary tract infections (UTI), daytime lower urinary tract (LUT) conditions, and urachal remnants (UR).

**Methods:** A structured literature assessment and comprehensive scoping exercise were performed to identify and appraise new evidence. Recommendations were developed by a panel based on the balance between desirable and undesirable consequences, evidence quality, and patient preferences. Clinical management algorithms were established for diagnosis, surgical intervention, and long-term follow-up.

**Results:** UDT affects 1.0–4.6% of full-term and 1.1–45% of preterm neonates. Laparoscopy for nonpalpable testes reveals anatomic findings including spermatic vessels entering the inguinal canal (40%), intra-abdominal (40%), or peeping (10%) testes, or vanishing testes (10%). Prepubertal testicular tumors have an incidence of 0.5 to 2 per 100,000 children, with 90% presenting as painless masses; recurrence after surgery was 5.8% (95% CI 2.3–14.1%). Hypospadias outcomes show success in 85–90% of distal and 32–86% of proximal cases, with complications in 23.3% of repeat and 12.2% of primary repairs. Fistula rates range from 5% to 50%. Inlay grafts reduce meatal stenosis risk by 66%. Glans dehiscence occurs in 9–17% of cases. CPC manifests as ventral (48%), lateral (24%), or dorsal (5%) deviation, with correction success rates of 90–100%. Daytime LUT conditions have a prevalence of 2% to 25%.

**Conclusion:** Surgical correction of UDT is highly recommended before 12 months, and by 18 months at the latest, to preserve fertility. Management of pediatric urological conditions requires early diagnosis, individualized surgical approaches, and structured transitional care starting between ages 12–16 years to ensure long-term health and independence.

***Abstract 15***

GPT5

Word count: 364

**Background**: To evaluate whether treatment with a combined antioxidant supplement improves semen parameters and pregnancy outcomes in men seeking fertility care compared with placebo.

**Methods**: This investigator-initiated, double-blind, placebo-controlled randomized clinical trial enrolled 1217 men aged 18 to 50 years across 21 centers. Participants were randomized 1:1 to receive an antioxidant supplement or placebo for 6 months. The primary outcome was ongoing pregnancy within 6 months after randomization, defined as a viable pregnancy at 12 weeks’ gestation. Secondary outcomes included biochemical pregnancy, clinical pregnancy, first-trimester pregnancy loss, ectopic pregnancy, cumulative pregnancy within 9 months, and time to pregnancy. Prespecified analysis assessed ongoing pregnancy between 4 and 6 months. Semen parameters and sperm DNA fragmentation were evaluated in subgroups. Analyses were conducted using an intention-to-treat framework with adjusted odds ratios (AORs) and 95% confidence intervals (CIs).

**Results**: A total of 1171 patients were included in the primary analysis (591 antioxidant; 580 placebo). Median male age was 34 (31–38) vs 35 (32–38) years, and female partner age was 32 (30–35) vs 33 (30–36) years. Ongoing pregnancy within 6 months occurred in 193/571 (33.8%) vs 208/555 (37.5%) (AOR, 0.85 [95% CI, 0.66–1.09]; P = .20). Between 4 and 6 months, ongoing pregnancy occurred in 69/446 (15.5%) vs 95/442 (21.5%) (AOR, 0.66 [95% CI, 0.47–0.94]; P = .02). Biochemical pregnancy was 244/571 (42.7%) vs 254/555 (45.8%) (AOR, 0.89 [95% CI, 0.70–1.13]; P = .35). Clinical pregnancy was 227/571 (39.8%) vs 240/555 (43.2%) (AOR, 0.87 [95% CI, 0.69–1.11]; P = .27). First-trimester loss was 58/571 (23.8%) vs 59/555 (23.2%) (AOR, 1.09 [95% CI, 0.71–1.68]; P = .69). Ectopic pregnancy was 5/571 (2.0%) vs 3/555 (1.2%) (AOR, 0.57 [95% CI, 0.13–2.39]; P = .44). Cumulative pregnancy was 0.61 (0.54–0.70) vs 0.66 (0.58–0.75) (RR, 0.93 [95% CI, 0.80–1.08]; P = .36), and time to pregnancy was 6.7 (6.4–6.9) vs 6.6 (6.4–6.9) months (HR, 0.94 [95% CI, 0.79–1.11]; P = .43).

**Conclusion**: In men seeking fertility care, treatment with a combined antioxidant supplement did not improve ongoing pregnancy rates and was associated with a significantly lower pregnancy rate within 4 to 6 months compared with placebo.

***Abstract 16***

Claude

Word count: 275

**Background:** To compare the efficacy and safety of Aquablation and laser enucleation of the prostate (LEP) for the treatment of lower urinary tract symptoms due to benign prostatic hyperplasia in men with prostate volumes between 80 and 180 ml.

**Methods:** WATER III (NCT04801381) was an investigator-initiated, prospective, randomized multicenter trial with concurrent nonrandomized cohorts conducted at three centers in Germany and two in the United Kingdom. Participants aged 45–80 years with an International Prostate Symptom Score (IPSS) ≥8 were enrolled. Randomized subjects were assigned 1:1 to Aquablation or LEP, stratified by center and aspirin use. The primary efficacy endpoint was the change in IPSS from baseline to 3 months, assessed using a Bayesian hierarchical model with a noninferiority margin of 4.7 points. The primary safety endpoint was the occurrence of Clavien-Dindo grade ≥2 adverse events.

**Results:** A total of 202 participants were enrolled, including 74 randomized and 128 in nonrandomized cohorts; 98 underwent Aquablation and 88 underwent LEP. At 3 months, the mean IPSS change was 12.9 ± 6.9 for Aquablation versus 13.1 ± 7.5 for LEP (posterior probability of noninferiority >0.999; posterior mean difference 0.93, 95% credible interval −1.48 to 3.53). Grade ≥2 complications occurred in 40.8% of Aquablation versus 56.8% of LEP patients (posterior mean difference −9.4%, 95% credible interval −31.8% to 12.9%). Persistent de novo ejaculatory dysfunction was lower after Aquablation (14.8%) than LEP (77.1%; p < 0.0001). LEP demonstrated superior Qmax improvement (p < 0.0001) and greater prostate volume reduction.

**Conclusion:** Aquablation demonstrated noninferior short-term symptom relief and comparable safety to LEP, with lower rates of ejaculatory dysfunction, supporting its role as an effective treatment alternative for large prostate glands.

***Abstract 17***

Human

Word count: 579

**Background:** Effective treatments are needed for patients with muscle-invasive bladder cancer scheduled for radical cystectomy who are ineligible for or decline to receive neoadjuvant cisplatin-based chemotherapy. We aimed to evaluate neoadjuvant TAR-200 plus cetrelimab (anti-PD-1) versus cetrelimab monotherapy in this setting.

**Methods:** SunRISe-4 is a randomised, open-label, phase 2 trial being conducted at 109 investigative centres in ten countries worldwide. Eligible patients were aged 18 years or older, were newly diagnosed with histologically confirmed muscle-invasive bladder cancer (stage cT2–cT4 N0M0), had an Eastern Cooperative Oncology Group performance status of 0–1, were scheduled to undergo radical cystectomy, and were deemed ineligible for or declined platinum-based neoadjuvant chemotherapy. Patients were randomly assigned (5:3) in blocks of eight using an interactive web response system to receive four cycles of intravesical TAR-200 (225 mg gemcitabine) plus intravenous cetrelimab (360 mg) every 21 days or four cycles of intravenous cetrelimab (360 mg) monotherapy every 21 days. Randomisation was stratified by results of transurethral resection of bladder tumour (visibly complete vs incomplete and ≤3 cm) and tumour stage (cT2 vs cT3–4a at initial diagnosis). The primary endpoint was centrally confirmed pathological complete response in the efficacy-evaluable set. As this was a prespecified interim analysis and all patients had not completed treatment, efficacy-evaluable set was defined as all patients who had radical cystectomy or progressive disease or death before radical cystectomy. Safety was analysed in all patients who received at least one dose of study drug. This trial is registered with ClinicalTrials.gov, NCT04919512, and is ongoing.

**Findings:** From July 7, 2022, to May 31, 2024, 196 patients were assessed for eligibility and 122 were randomly assigned (TAR-200 plus cetrelimab n=80, cetrelimab monotherapy n=42). 120 patients received at least one dose of study drug. Mean age was 70·7 years (SD 7·9); 102 (85%) participants were male, 18 (15%) were female, 81 (68%) were White, 28 (23%) were Asian, and 11 (9%) were other races. In the efficacy-evaluable set (TAR-200 plus cetrelimab n=53, cetrelimab monotherapy n=31), at a median follow up of 23·5 weeks (IQR 8·6–42·0), pathological complete response rates were 42% (22 of 53 patients; 95% CI 28–56) in the TAR-200 plus cetrelimab cohort and 23% (seven of 31 patients; 10–41) in the cetrelimab monotherapy cohort. In the safety set, at a median follow-up of 10·2 weeks (IQR 1·1–36·9), treatment-related adverse events occurred in 57 (72%) of 79 patients in the TAR-200 plus cetrelimab cohort and in 18 (44%) of 41 patients in the cetrelimab monotherapy cohort. Grade 3 or worse treatment-related adverse events occurred in nine (11%) patients in the TAR-200 plus cetrelimab cohort and two (5%) in the cetrelimab monotherapy cohort, the most common being haematuria (two [3%] in the TAR-200 plus cetrelimab cohort). Serious treatment- related adverse events occurred in nine (11%) patients in the TAR-200 plus cetrelimab cohort and one (2%) patient in the cetrelimab monotherapy cohort. In the TAR-200 plus cetrelimab cohort, seven (9%) patients had treatment- related adverse events leading to discontinuation of TAR-200 and six (8%) had treatment-related adverse events leading to discontinuation of cetrelimab; there were no treatment related deaths. In the cetrelimab monotherapy cohort, no patients discontinued due to treatment-related adverse events; there was one death from a treatment-related adverse event due to hyperglycaemic, hyperosmolar, non-ketotic syndrome.

**Conclusions:** Neoadjuvant TAR-200 plus cetrelimab showed a high pathological complete response rate with a manageable safety profile. These results support continued investigation of TAR-200 in patients with muscle-invasive bladder cancer planned for radical cystectomy.

***Abstract 18***

GPT5

Word count: 361

**Background:** To evaluate whether metastasis-directed therapy (MDT) added to androgen deprivation therapy (ADT) improves outcomes in oligometastatic prostate cancer and to assess associated systemic immune responses.

**Methods:** EXTEND was a phase 2, open-label, multicenter basket trial in men aged at least 18 years with 1–5 metastases, Eastern Cooperative Oncology Group performance status 0–2, and 4 or fewer prior systemic therapy lines. Patients were randomized 1:1 to MDT plus intermittent or continuous ADT versus ADT alone. The primary endpoint was progression-free survival (PFS). Secondary endpoints included radiologic PFS (rPFS), castration resistance–free survival (CRFS), time to new lesion, time to next-line systemic therapy, overall survival, safety, and translational immune correlatives.

**Results:** Of 265 screened patients, 183 were randomized and 174 were evaluable: MDT+cADT, n=45; cADT, n=42; MDT+iADT, n=43; iADT, n=44. ARPC was present in 41/174 (24%), including 34/87 (39%) in the cADT basket. Staging used conventional imaging in 102/174 (59%), fluciclovine PET in 50/174 (29%), and PSMA PET in 22/174 (13%). ARPI was received by 95/174 (55%), including 67/133 (50%) with omHSPC and 28/41 (68%) with omARPC. In the MDT arm, 88 patients received definitive radiotherapy to 196/196 (100%) metastases. Median follow-up was 31 months for cADT, 47 months for iADT, and 42 months for combined analyses. Median PFS was 47 versus 22 months in cADT (HR, 0.50; 95% CI, 0.23–1.08; p=0.036), 28 versus 16 months in iADT (HR, 0.44; 95% CI, 0.25–0.78; p=0.005), and 36 versus 17 months combined (HR, 0.45; 95% CI, 0.30–0.69; p<0.001). Combined rPFS HR was 0.63 (95% CI, 0.40–0.97; p=0.04), CRFS HR was 0.40 (95% CI, 0.19–0.82; p=0.01), and OS HR was 0.54 (95% CI, 0.24–1.21; p=0.13). Four patients (4.5%) had local failure. No grade 4 or 5 toxicities occurred. T-cell receptor repertoire modulation was associated with improved PFS (HR, 0.21; 95% CI, 0.06–0.76; p=0.02), adjusted median PFS 60 versus 28 months, and restricted mean PFS gain 11 months (95% CI, 2–20; p=0.02).

**Conclusion:** In oligometastatic prostate cancer, adding MDT to ADT improved PFS, rPFS, and CRFS, with no grade 4 or 5 toxicities reported. Immune correlatives linked T-cell receptor repertoire modulation after MDT+ADT with longer PFS.

***Abstract 19***

Claude

Word count: 324

**Background:** Bowel and bladder dysfunction (BBD) affects up to 40% of pediatric urology patients, yet a significant subset remains refractory to first-line urotherapy. No study has explored whether initial demographic and symptom data can predict early treatment non-response in this population. This study aimed to develop a predictive model using demographic and questionnaire-derived clinical features to identify pediatric BBD patients at high risk for non-response to standard urotherapy.

**Methods:** This retrospective cohort study included pediatric BBD patients aged 5–10 years evaluated between 2020 and 2023 at the University of Iowa Pediatric Urology Clinic. Symptom profiles were collected using a validated 18-item BBD questionnaire, with responses dichotomized for analysis. The primary outcome was non-response to urotherapy, defined as an unchanged or worsening follow-up symptom score. Multivariable logistic regression and random forest models were developed using 15 predictors with 80/20 holdout validation. Model performance was assessed by area under the receiver-operating-characteristic curve (AUROC), calibration, and decision curve analysis.

**Results:** A total of 123 patients met inclusion criteria, with a median age of 6 years (IQR 5, 8); 92 (75%) were female. The median baseline symptom score was 4 (IQR 3, 5) and follow-up score was 2 (IQR 1, 4). Complete improvement occurred in 26 (21%) patients, moderate in 28 (23%), minimal in 23 (19%), and no improvement in 46 (38%). On multivariable logistic regression, older age (OR 1.45, 95% CI 1.09–1.98; p=0.01) and dysuria (OR 1.54, 95% CI 1.06–2.37; p=0.03) were associated with treatment response, while daytime incontinence (OR 0.67, 95% CI 0.46–0.97; p=0.04) predicted non-response. The logistic regression model achieved an AUROC of 0.67; the random forest model achieved an AUROC of 0.71.

**Conclusion:** Demographic and symptom-based predictors derived from standardized questionnaires can identify pediatric BBD patients at risk for urotherapy non-response. Key predictors included age, dysuria, and daytime incontinence. While machine learning modestly enhanced predictive accuracy over traditional regression, clinical utility remains preliminary and requires external validation across diverse cohorts.

***Abstract 20***

GPT5

Word count: 250

**Background:** To summarize evidence on evaluation and treatment of erectile dysfunction.

**Methods:** An International Consensus of Sexual Medicine committee reviewed the literature, discussed recommendations in Madrid in July 2024, and finalized guidance using a modified Delphi method.

**Results:** Committee size was not numerically reported; reviewed populations included men with vasculogenic, neurogenic, endocrine, metabolic, cardiovascular, renal, and spinal cord injury-related erectile dysfunction. Erectile dysfunction prevalence was 66% in type 2 diabetes and 37.5% in type 1 diabetes. Spinal cord injury incidence was 250000-500000 annually, 78% of patients were male, mean age was 43 years, and among 774 patients, 13% of quadriplegic and 27% of paraplegic respondents rated improved sexual function a top priority. Placebo response in erectile dysfunction drug trials was 25%-35%. In FM57, 250 participants from 9 European countries used Eroxon for 12 weeks; 61% with mild, 59% with moderate, and 80% with severe erectile dysfunction achieved or exceeded the minimum clinically important difference, with headaches in 3% and localized burning in 1%. Botulinum toxin trials enrolled 70 and 176 patients; 53% versus 1 control patient achieved sufficient erection in the smaller trial, and approximately 40% responded in the larger trial, with a significant difference favoring BTX-100 at 6 months (P < .01). In 1493 hypogonadal men, testosterone injection reduced phosphodiesterase-5 inhibitor nonresponse from 25% to 12% and increased satisfactory response from 35% to 57%.

**Conclusion:** Erectile dysfunction management should integrate evaluation, risk-factor modification, phosphodiesterase-5 inhibitors, testosterone replacement in hypogonadal men, and escalation to devices, intracavernosal therapy, prostheses, or treatment.

***Abstract 21***

Claude

Word count: 273

**Background:** To evaluate the effectiveness of a patient-oriented online decision aid compared to a printed brochure for men with nonmetastatic prostate cancer, with a primary focus on guideline-conforming use of active surveillance or watchful waiting (observation) as the final treatment decision.

**Methods:** A randomized controlled prospective trial with parallel and equal group allocation was conducted across 116 urological practices and clinics throughout Germany from April 1, 2018 to December 31, 2021. Male patients aged 18–80 years with histologically confirmed nonmetastatic adenocarcinoma of the prostate were randomized 1:1 to receive either an online decision aid or a printed patient guideline brochure. The primary outcome was treatment decision at 14 months. Seven secondary outcomes were assessed using validated questionnaires at baseline, 4-week, and 14-month follow-up.

**Results:** Data from 1,000 patients were available for final analysis (intervention n = 516; control n = 484). Baseline parameters were comparable between groups. No statistically significant difference in the proportion of observation was found between groups overall (intervention 15.3% vs. control 17.4%, p = 0.4), at low/early-intermediate risk (39.7% vs. 41.1%, p = 0.8), or at intermediate/high risk (4.2% vs. 6.1%, p = 0.3). Among secondary endpoints, satisfaction with involvement in decision-making at T2 showed superiority of the intervention group (90.6% vs. 86.2%, p = 0.044). Follow-up response rate was 9% lower in the intervention group (83.1% vs. 92.1%, p < 0.001).

**Conclusion:** The online decision aid demonstrated comparable effectiveness to the printed brochure regarding the primary endpoint of guideline-conforming observation rates. Structural improvements in the counseling process were observed, including one significant secondary outcome favoring the intervention, though the primary and remaining secondary hypotheses were not confirmed.

***Abstract 22***

Human

Word count: 240

**Background:** To present large-scale safety outcomes, with a particular focus on postoperative bleeding following Aquablation for benign prostatic hyperplasia.

**Methods:** Patients who underwent Aquablation between 2019 and 2024 across Asia, Europe, and North America were assessed to evaluate trends in treated prostate sizes, which were visualised using density plots. A corporate prospective database was maintained, incorporating case recordings and data collected by on-site company representatives. In addition, the incidence of postoperative bleeding-defined as transfusion or surgical takeback for haemostatic fulguration-was analysed using data from the United States Food and Drug Administration (FDA) Manufacturer and User Facility Device Experience (MAUDE) database and procedure counts by the manufacturer.

**Results:** A total of 70 270 Aquablation procedures were evaluated over the period from 2019 to 2024. The mean (standard deviation) prostate volume was 87.3 (42.4) mL, with a maximum recorded size of 1189 mL. Density plot analysis of prostate volumes demonstrated consistent utilisation of Aquablation across the full range of prostate sizes throughout all years studied. The overall rate of blood transfusion or return to the operating room for haemostatic fulguration was 0.2%, indicating a favourable safety profile across a very wide range of prostate sizes.

**Conclusions:** Aquablation has been consistently utilised across a broad spectrum of prostate sizes, with a low overall rate of transfusion or return to the operating room for bleeding control. These findings highlight the procedure's broad applicability and favourable safety profile in real-world practice from 2019 to 2024.

***Abstract 23***

Human

Word count: 352

**Background**: The optimal patient position for percutaneous nephrolithotomy (PCNL) remains a matter of debate. Our aim was to evaluate the association between prone versus supine positioning and perioperative and postoperative outcomes of suction mini-PCNL.

**Methods:** In this prospective multicenter observational study, we analyzed data for 1534 patients treated in 30 centers between March and November 2024. Outcomes included the stone-free rate (SFR) assessed via 30-d computed tomography, and complication rates. Multivariable analysis was used to assess the effect of prone positioning on stone-free status (zero fragments) and overall complications, with adjustment for other covariates.

**Results**: There were 653 patients (43%) in the prone group and 881 (57%) in the supine group. Patient demographics were similar between the groups, except for body mass index. In terms of Guy’s stone score, the prone group had a higher proportion of score 1 stones (60% vs 47%) and the supine group a higher proportion of score 4 stones (6.9% vs 3.2%). Median stone volume did not differ significantly, at 1636 mm3 in the supine group and 1725 mm3 in the prone group (p = 0.7). The prone group had more frequent use of spinal anesthesia (68% vs 29%; p < 0.001), fluoroscopy-only guidance (86% vs 61%; p < 0.001), and supracostal access (36% vs 22%; p < 0.001). Surgical time, pain scores, hospital length of stay, and readmission rates were similar between the groups. Zero-fragment stone-free rates were comparable (85% prone vs 81% supine; p = 0.052). Prone position was associated with higher rates of blood transfusion (2.8% vs 0%; p < 0.001), renal pelvic perforation (2.8% vs 0.23%; p < 0.001), and pneumothorax (1.5% vs 0%; p < 0.001). Multivariable analysis revealed that prone positioning was not significantly associated with grade A stone-free status (odds ratio 0.92, 95% confidence interval [CI] 0.66–1.29; p = 0.6) or the overall complication rate (odd ratio 0.87, 95% CI 0.59–1.28; p = 0.5). The nonrandomized study design may have introduced selection bias and limited our ability to establish causal relationships between variables.

**Conclusion:** Both prone and supine positioning for PCNL achieved excellent SFRs with acceptable safety profiles.

***Abstract 24***

Claude

Word count: 307

**Background:** Platelet-rich plasma (PRP) has emerged as a regenerative treatment for erectile dysfunction (ED), yet its efficacy remains unclear. This systematic review and meta-analysis aimed to summarize and critically evaluate all high-quality randomized controlled trials (RCTs) assessing the effect of PRP injections on erectile function in men with ED.

**Methods:** A comprehensive literature search was conducted across PubMed, Google Scholar, EMBASE, the Cochrane Library, Scopus, and Web of Science. Only RCTs were included. Studies involving ejaculatory or other sexual dysfunctions, or those combining PRP with other regenerative or non-regenerative therapies, were excluded. Methodological quality was assessed using the Revised Cochrane Risk of Bias 2 tool, and evidence certainty was graded using the GRADE approach. Meta-analysis of standardized mean differences (SMD) in International Index of Erectile Function (IIEF) scores was performed at 1, 3, and 6 months using a random-effects model.

**Results:** Seven RCTs comprising 512 participants were included, with sample sizes ranging from 52 to 120. PRP injection volumes ranged from 5 to 10 cc, administered across 2 to 4 sessions spaced 2 to 4 weeks apart. At 1 month, the pooled SMD was 0.28 (95% CI: −0.11 to 0.67, P = .16). At 3 months, the pooled SMD was 0.29 (95% CI: −0.08 to 0.67). At 6 months, the pooled SMD was 0.36 (95% CI: −0.09 to 0.81, P = .12). Heterogeneity was substantial across all time points (I² = 77–83%, all P < .01). No statistically significant improvement in IIEF scores was demonstrated at any follow-up interval.

**Conclusion:** This meta-analysis of 7 RCTs found no statistically significant benefit of PRP injections over placebo in improving erectile function at 1, 3, or 6 months. High heterogeneity across studies limits the reliability of these findings. Large-scale, standardized RCTs with rigorous protocols and longer follow-up are required to definitively establish the role of PRP in ED management.

***Abstract 25***

GPT5

Word count: 263

**Background:** To conduct an in-depth analysis of post-Aquablation bleeding and assess real-world utilization patterns using global data, with a focus on temporal trends in transfusion and haemostatic intervention rates across diverse practice settings and prostate sizes.

**Methods:** A prospective database incorporating data from on-site company representatives and procedural recordings was analysed alongside the FDA Manufacturer and User Facility Device Experience database. Data included procedure date, prostate size, and reported adverse events across multiple countries. Patients treated between 2019 and 2024 in Asia, Europe, and North America were evaluated. Outcomes included transfusion and takeback for cautery, with prostate size distributions assessed using density plots and stratified regional analyses.

**Results:** A total of 70,270 Aquablation procedures were performed between 2019 and 2024. The mean prostate volume was 87.3 mL with a standard deviation of 42.4 mL, and a maximum volume of 1189 mL. Transfusion rates decreased from 2.3% in 2019 to 0.1% in 2023 and 2024, with an overall pooled incidence of 0.2%. Takeback for cautery declined from 2.7% in 2019 to 0.1% between 2022 and 2024, also yielding an overall rate of 0.2%. Procedure counts increased annually from 964 in 2019 to 33,716 in 2024. Regional analysis showed 46.1% of prostates >80 mL in North America, while Europe/Middle East had 1.6% <30 mL. Reported transfusion rates were 0.2% in North America and Europe/Middle East and 0.1% in Asia.

**Conclusion:** This large-scale analysis demonstrates increasing global adoption of Aquablation from 2019 to 2024, with consistently low and decreasing transfusion and takeback rates, supporting a favourable perioperative safety profile across a wide range of prostate sizes.

***Abstract 26***

Claude

Word count: 270

**Background:** To compare oncological and functional outcomes between three-dimensional artificial intelligence–augmented reality (3D-AI-AR) guided robot-assisted radical prostatectomy (RARP) and standard two-dimensional MRI-guided RARP in patients with locally advanced prostate cancer exhibiting extracapsular extension (ECE) or bulging on preoperative MRI.

**Methods:** This prospective, multicenter, two-arm randomized controlled trial enrolled adults aged ≥40 years with monolateral localized or locally advanced prostate cancer showing ECE or bulging on preoperative MRI (PIRADS ≥3). Patients were randomized in a 1:2 ratio (3D-AI-AR:MRI) with stratification by age, PSA density, prostate volume, lesion volume, and ECE/bulging presence. The primary outcome was positive surgical margin rate (PSMr). Secondary outcomes included continence recovery, potency recovery, and biochemical recurrence. Statistical analyses included Fisher's exact test, odds ratios with 95% confidence intervals, and Kaplan-Meier survival analysis.

**Results:** Of 138 patients assessed, 136 were randomized: 52 to the 3D-AR group (49 analyzed) and 84 to the MRI group. PSMs occurred in 23/49 (47%) in the 3D-AR group versus 38/84 (45%) in the MRI group (p = 0.8). PSMs at the neurovascular bundle level were significantly lower in the 3D-AR group (12/23, 52%) versus the MRI group (5/38, 13%; p = 0.001). After excisional biopsies, PSMs on preserved bundles were 11/49 (22%) versus 33/84 (39%) for the 3D-AR versus MRI groups (p = 0.047). Social continence at 12 months was 94% versus 75% (p = 0.03; OR 0.9, 95% CI 0.4–2.0). Potency recovery at 12 months was comparable (p = 0.3).

**Conclusion:** 3D-AI-AR guidance during nerve-sparing RARP significantly reduced positive surgical margins at preserved neurovascular bundles and improved 12-month social continence compared with standard MRI-guided surgery, without compromising overall oncological outcomes.

***Abstract 27***

GPT5

Word count: 260

**Background:** To assess neoadjuvant TAR-200 plus cetrelimab versus cetrelimab monotherapy in muscle-invasive bladder cancer among patients ineligible for or declining platinum-based neoadjuvant chemotherapy.

**Methods:** SunRISe-4 is an ongoing, randomised, open-label, phase 2 trial. Adults with cT2–cT4 N0M0 disease and ECOG performance status 0–1 were randomly assigned 5:3 to TAR-200 plus cetrelimab or cetrelimab alone. TAR-200 delivered 225 mg intravesical gemcitabine every 21 days for four cycles; cetrelimab 360 mg was given intravenously every 21 days for four cycles. The primary endpoint was centrally confirmed pathological complete response.

**Results:** From July 7, 2022, to May 31, 2024, 196 patients were assessed, 122 were randomly assigned, and 120 comprised the safety population: 79 received TAR-200 plus cetrelimab and 41 received cetrelimab monotherapy. Mean age was 70.7 years and 102 (85%) were male. The efficacy-evaluable population included 53 and 31 patients. Pathological complete response was 42% (22/53; 95% CI 28–56) versus 23% (7/31; 10–41), and pathological overall response was 60% (32/53; 46–74) versus 35% (11/31; 19–55). In cT2 disease, pathological complete response was 48% (19/40; 32–64) versus 23% (6/26; 9–44). Median time to radical cystectomy was 13.7 weeks (IQR 12.7–14.6) versus 12.6 weeks (12.0–14.4). Treatment-related adverse events occurred in 57/79 (72%) versus 18/41 (44%), with grade 3 or worse events in 9/79 (11%) versus 2/41 (5%).

**Conclusion:** In this interim analysis, TAR-200 plus cetrelimab showed higher complete and overall response rates than cetrelimab monotherapy, with grade 3 or worse adverse events in 11% and 5%, supporting continued investigation in localised muscle-invasive bladder cancer.

***Abstract 28***

Claude

Word count: 287

**Background:** To evaluate the efficacy of metastasis-directed therapy (MDT) combined with androgen deprivation therapy (ADT) versus ADT alone on progression-free survival (PFS) and systemic antitumor immune responses in patients with oligometastatic prostate cancer (omPC).

**Methods:** EXTEND (NCT03599765) is a phase 2, open-label, multicenter, randomized basket trial enrolling men aged ≥18 years with one to five omPC metastases and an Eastern Cooperative Group performance status of 0–2. Patients were randomized 1:1 to MDT plus ADT or ADT alone. The primary endpoint was PFS; secondary endpoints included radiologic PFS (rPFS), castration resistance–free survival (CRFS), and overall survival (OS). Translational analyses included longitudinal profiling of T-cell receptor (TCR) clonal expansion and contraction. Significance was set at p < 0.10 (one-sided) for the primary endpoint.

**Results:** From September 2018 through August 2022, 265 patients were screened and 183 randomized; 174 were eligible for per-protocol primary endpoint analysis. In the combined analysis (median follow-up 42 months), MDT plus ADT significantly improved PFS (HR, 0.45; 95% CI, 0.30–0.69; p < 0.001), with a median PFS of 36 months versus 17 months for ADT alone. rPFS was also improved (HR, 0.63; 95% CI, 0.40–0.97; p = 0.038). CRFS was superior in the MDT arm among omHSPC patients (HR, 0.40; 95% CI, 0.19–0.82; p = 0.01). TCR repertoire modulation after MDT plus ADT was associated with improved PFS (HR, 0.21; 95% CI, 0.06–0.76; p = 0.02), with an adjusted median PFS of 60 months versus 28 months.

**Conclusion:** MDT plus ADT significantly improved PFS, rPFS, and CRFS in omPC patients, delaying progression to castration-resistant disease. These clinical benefits may be partially mediated by induction of systemic antitumor immune responses, supporting further investigation of MDT combined with T-cell–targeted immunotherapies.

***Abstract 29***

GPT5

Word count: 253

**Background:** To evaluate whether an online decision aid improves treatment decision-making versus a printed brochure in men with nonmetastatic prostate cancer.

**Methods:** In this randomized trial, men aged 18–80 years with nonmetastatic prostate adenocarcinoma were recruited at 116 urological practices and clinics and randomized 1:1 to an online decision aid or a printed brochure. The primary endpoint was treatment decision after 14 months, with observation defined as active surveillance or watchful waiting.

**Results:** Of 1115 randomized patients, 559 were assigned to the intervention group and 556 to the control group; 1000 patients were available for final analysis. Response was lower in the intervention group than in controls (83.1% vs 92.1%, p < 0.001). Final treatment decisions were radical prostatectomy in 59%, radiotherapy in 16%, observation in 16%, and other in 9%. Observation did not differ overall (15.3% vs 17.4%, p = 0.4), in low/early-intermediate risk disease (39.7% vs 41.1%; difference, 1.5%; 95% CI, −13.4% to 10.5%), or in intermediate/high-risk disease (4.2% vs 6.1%; difference, 1.9%; 95% CI, −1.7% to 5.6%). Secondary outcomes showed no significant differences except satisfaction with the doctor’s involvement at T2 (90.6% vs 86.2%, p = 0.044). Exploratory analyses favored the intervention for bringing a preparation summary (75.8% vs 56.0%, p < 0.001), shorter consultation time (61.1 ± 45.2 vs 69.4 ± 55.6 minutes, p = 0.02), and more discussion of radiotherapy (90.1% vs 83.6%, p = 0.005).

**Conclusion:** The online decision aid did not improve the primary outcome or most secondary outcomes, although exploratory measures favored the intervention.

***Abstract 30***

Gemini

Word count: 267

**Background:** To assess whether treatment of men seeking fertility care with a combined antioxidant supplement improves semen quality and pregnancy rates compared with a placebo.

**Methods:** This double-blind, placebo-controlled randomized clinical trial, the SUMMER trial, was conducted from May 2018 to February 2024 across 21 centers in the Netherlands. Participants included men aged 18 to 50 years with female partners aged 18 to 43 years experiencing infertility. Men were randomized 1:1 to receive either a daily combined antioxidant supplement or a placebo for six months. The primary outcome was ongoing pregnancy conceived within six months after randomization. Secondary outcomes included biochemical pregnancy rates, semen parameters, and IVF or ICSI outcomes.

**Results:** A total of 1217 patients were randomized; 1171 were included in the primary analysis (591 antioxidant group, 580 placebo). The ongoing pregnancy rate within six months was 33.8% (193/571) in the antioxidant group versus 37.5% (208/555) in the placebo group (AOR 0.85 [95% CI, 0.66–1.09]; P=.20). Between four and six months, the antioxidant group had a significantly lower ongoing pregnancy rate of 15.5% (69/446) compared to 21.5% (95/442) for placebo (AOR 0.66 [95% CI, 0.47–0.94]; P=.02). For IVF/ICSI, pregnancy after fresh embryo transfer was 43.2% (67/156) in the antioxidant group and 56.2% (82/146) in the placebo group (AOR 0.53 [95% CI, 0.33–0.85]; P=.009). Treatment adherence was 58.0%. Adverse events occurred in 2.7% (16/591) and 1.7% (10/580) of patients, respectively (P=.29).

**Conclusion:** Treatment with combined antioxidant supplements does not improve ongoing pregnancy rates or semen parameters in men seeking fertility care. These findings do not support the use of such supplements for these patients.

***Abstract 31***

Human

Word count: 296

**Background:** The issue of sexual dysfunction in infertile couples is often neglected and underreported. As sexual dysfunction can both contribute to and result from infertility, clinicians should be equipped to identify and address these issues as part of comprehensive fertility care. To develop evidence- and consensus-based recommendations for the clinical management of male sexual dysfunction (MSD) in the context of infertility.

**Methods:** Initial recommendations were formulated based on expert opinion and exploratory analysis of various types of MSD associated with infertility. A focused literature review was conducted for each topic, followed by iterative rounds of expert discussion to refine recommendations. Final consensus was achieved at the 5th International Consultation on Sexual Medicine meeting, and recommendations were rated using GRADE criteria.

**Results:** MSD and infertility often coexist, necessitating a detailed sexual history and physical examination during the initial infertility evaluation. Erectile dysfunction may be effectively managed with counseling, phosphodiesterase-5 inhibitors, or intracavernosal injections (eg, alprostadil, papaverine, phentolamine), which do not impair fertility outcomes. For low libido or unconsummated marriages, a multidisciplinary approach tailored to the couple’s priorities—sexual function or fertility—is recommended. Ejaculatory disorders may be treated with counseling, penile vibratory stimulation, electro-ejaculation, medications, or assisted reproduction, depending upon the underlying cause. Selective serotonin reuptake inhibitors, used for premature ejaculation, may adversely affect sperm parameters and should be prescribed cautiously. Men with hypogonadism seeking fertility should avoid exogenous testosterone; alternatives such as selective estrogen receptor modulators, aromatase inhibitors, or gonadotropins may be considered. Lifestyle optimization, management of comorbidities, and use of fertility-safe lubricants can improve sexual and reproductive outcomes for couples trying to conceive.

Conclusion: MSD and infertility are often interrelated. Incorporating routine sexual health assessments into fertility evaluations enables clinicians to diagnose and treat MSD effectively, thereby i mproving both sexual function and reproductive success.

***Abstract 32***

GPT5

Word count: 250

**Background:** To evaluate intravaginal electrical stimulation (IVES) as a nonsurgical treatment for urinary incontinence in women.

**Methods:** This systematic review followed PRISMA and was registered in PROSPERO (CRD42022318840). PubMed, EMBASE, Ovid Medline, and IEEE Xplore were searched through 9 November 2020, with updates on 7 August 2023 and 7 February 2025. English-language randomized trials were included. Risk of bias was assessed with ROB-2. Meta-analysis was not performed because of heterogeneity.

**Results:** Among 4,898 records identified, 1,656 duplicates were removed, 3,242 records were screened, 193 full-text reports were assessed, 161 were excluded, and 32 randomized controlled trials were included. Thirty-one trials (96.9%) enrolled general populations of women and 1 enrolled postpartum women. UI subtypes included stress UI (n=19), mixed UI (n=10), urgency UI (n=14), and unspecified UI (n=3). Combined interventions were used in 9 of 32 trials (28.1%). Significant within-group improvement after IVES was reported in 6 of 8 trials (75%) for pad number, 10 of 15 (66.7%) for pad weight, 8 of 14 (57.1%) for leakage episodes, and 11 of 19 for pelvic floor muscle strength. Of 27 quality-of-life trials, 19 (70.4%) reported improvement and 15 of 19 (78.9%) showed statistically significant within-group differences. Adherence ranged from 61% to 100%. Twenty trials assessed adverse events, and frequency was low.

**Conclusion:** IVES may be a safe, acceptable option for women with UI, with moderate-to-high adherence and benefits for leakage-related outcomes, pelvic floor muscle strength, and quality of life. However, low-quality, heterogeneous evidence prevents conclusions regarding efficacy or superiority over conservative treatments.

***Abstract 33***

Claude

Word count: 277

**Background:** To assess the safety and feasibility of stone extraction using the hydrogel method (HM) with mediNiK® (MN) compared with standard of care during flexible ureterorenoscopy (fURS) with lithotripsy in patients with kidney stones larger than 8 mm.

**Methods:** This single-blinded, prospective, multicenter, randomized study was conducted between September 2021 and September 2022 across five German hospitals. Seventy patients scheduled for elective fURS with lithotripsy were screened and randomized to fURS + MN or fURS alone. Primary objectives were feasibility and safety, assessed via the number of extracted fragments stratified by size (<0.5 mm, 0.5–1 mm, >1 mm), treatment-emergent adverse events (TEAEs), operative duration, and surgeon-rated difficulty on a 5-point Likert scale. Statistical analyses used Student's t-test, Mann–Whitney U test, and Chi-square test.

**Results:** Of 70 screened patients, 65 were randomized and 58 received the allocated intervention (n=29 per arm; Safety Evaluation Set). Baseline characteristics were comparable (fURS + MN: mean age 52.3 ± 15.9 years, BMI 27.6 ± 4.44; fURS alone: 51.7 ± 16.5 years, BMI 28.6 ± 6.74; 34.5% female in both arms). TEAEs occurred in 17.2% (n=5) of fURS + MN versus 24.1% (n=7) of fURS alone patients, with no severe events. fURS + MN yielded approximately 8-fold more fragments <1 mm (1716 vs 209) and twice as many fragments >1 mm (310 vs 118). Median fragments per patient were 7 (IQR 21) versus 0 (IQR 2), p=0.013. Median operative time was longer with fURS + MN (80 vs 62 min, p=0.02), without difference in perceived difficulty (p=0.29).

**Conclusion:** HM during fURS was safe and feasible, facilitating retrieval of small fragments that might otherwise be missed with standard techniques, without increasing complications.

***Abstract 34***

Gemini

Word count: 256

**Background:** This systematic review and meta-analysis aimed to summarize and critically evaluate high-quality randomized controlled trials (RCTs) assessing the efficacy and safety of platelet-rich plasma (PRP) injections for the treatment of erectile dysfunction (ED).

**Methods:** The study was conducted according to PRISMA guidelines and registered in PROSPERO. A comprehensive search across multiple electronic databases identified 7 eligible RCTs comparing PRP monotherapy against placebo or control groups. Standardized mean differences (SMD) and 95% confidence intervals (CI) were calculated using a random-effects model to account for study heterogeneity. Quality of evidence was assessed via the Cochrane Risk of Bias 2 tool and the GRADE approach.

**Results:** Across 7 RCTs, 512 participants were included with sample sizes ranging from 52 to 120. PRP injection volumes varied between 5 and 10 cc. At 1-month follow-up, the pooled SMD was 0.28 (95% CI: -0.11 to 0.67, P = .16). At 3 months, the SMD was 0.29 (95% CI: -0.08 to 0.67), suggesting small but statistically significant improvement. By 6 months, the pooled SMD was 0.36 (95% CI: -0.09 to 0.81, P = .12). Heterogeneity remained high (I² > 70%) across all intervals. Adverse events were rare, with only one study reporting a single case each of hematoma and plaque formation.

**Conclusion:** Current evidence regarding PRP for ED is mixed, demonstrating only marginal, statistically non-significant improvements in erectile function at most time points. Significant methodological heterogeneity and risk of bias limit the certainty of these findings. Large-scale RCTs with standardized protocols are essential before PRP can be recommended for routine clinical use.

***Abstract 35***

Human

Word count: 311

**Background:** Three-dimensional (3D) augmented reality (AR) and artificial intelligence (AI) technologies have recently been introduced to enhance guidance during robot-assisted radical prostatectomy (RARP). By overlaying virtual and real-time images, this approach helps accurately localize hidden lesions during surgery, enabling the execution of tailored procedures. This study aimed to evaluate whether 3D-AI-AR guidance reduces positive surgical margins (PSMs) compared with standard two-dimensional (2D) magnetic resonance imaging (MRI)-based interventions.

**Methods:** In this prospective, multicenter randomized controlled trial (NCT06318559), 133 patients with extracapsular extension or bulging at preoperative MRI were enrolled and randomized (2:1) to either 2D MRI-guided (n = 84) or 3D-AI-AR–guided RARP (n = 49). All patients underwent nerve-sparing RARP. Intraoperative selective biopsies were then performed at the level of the preserved neurovascular bundle (NVB): cognitive in the MRI group and AR guided in the 3D group. The primary outcome was PSM rate. Prostate-specific antigen (PSA) levels, continence, and potency recovery were assessed during 12 months of follow-up. The use of postoperative radiotherapy was recorded. Biochemical recurrence (BCR) was defined as PSA >0.4 ng/ml. All analyses were conducted with SAS Statistics Software v.9.4.

**Results:** Baseline and intraoperative characteristics were similar between the groups. While PSMs on the prostate surface were comparable (p = 0.8), 3D-guided excisional biopsies had a significantly higher positivity rate (52% vs 13%; p = 0.001), allowing improved margin control. The 3D group had a lower overall PSM rate (22% vs 39%; p = 0.047), required less postoperative radiotherapy (18% vs 35%; p = 0.046), and showed higher continence at 12 months (91% vs 71%; p = 0.03). Potency and BCR rates were similar.

**Conclusion:** The execution of a 3D-AI-AR–guided biopsy at the level of preserved NVBs during nerve-sparing RARP allows correct identification of the tumor with subsequent improvement of margin control. Longer follow-up is required to assess the functional and long-term oncological outcomes of this approach.

***Abstract 36***

Gemini

Word count: 271

**Background:** The primary objective of this prospective multicenter study was to evaluate the safety and feasibility of the hydrogel method using mediNiK as an adjunct to flexible ureterorenoscopy for the extraction of kidney stone fragments. The study aimed to compare the efficacy of gel-assisted fragment removal against the standard of care in achieving stone clearance.

**Methods:** This single-blinded randomized study involved seventy patients at five German hospitals between September 2021 and September 2022. Inclusion required stones larger than 8 mm. Patients were randomized to receive either flexible ureterorenoscopy alone or flexible ureterorenoscopy combined with mediNiK, a biocompatible hydrogel comprising two liquid components (K1-Alginate and K2-Calcium ions). Extracted fragments were size-fractionated into categories of less than 0.5 mm, 0.5 to 1 mm, and 1 mm or greater for quantitative analysis.

**Results:** The safety evaluation set included 58 patients (n=29 per arm), with a mean age of 52.3 years in the mediNiK group and 51.7 years in the control group. Treatment-emergent adverse events occurred in 17.24% (5/29) of the mediNiK group versus 24.14% (7/29) in the control group. MediNiK resulted in an 8-fold higher extraction of fragments smaller than 1 mm (1716 versus 209 fragments). A median of 7 fragments was extracted per patient with mediNiK versus 0 in the control group (p=0.013). Median procedural time was significantly longer for mediNiK at 80 minutes compared to 62 minutes (p=0.02), though subjective difficulty showed no significant difference (p=0.29).

**Conclusion:** Hydrogel-assisted extraction during flexible ureterorenoscopy is safe and feasible, significantly increasing the retrieval of small stone fragments. Although the procedure duration is longer, the method effectively targets submillimeter debris that typically remains as residual fragments.

***Abstract 37***

Human

Word count: 256

**Background:** We present a summary of part II of the 2024 update of the European Association of Urology (EAU)/European Society of Paediatric Urology (ESPU) guidelines on paediatric urology. The summary provides evidence-based standards for management of a number of urological conditions in the paediatric population. The aim is to provide practical recommendations for clinical management of these conditions with a focus on diagnosis, treatment, and follow-up.

**Methods:** For the guidelines update, new and relevant evidence was identified, collated, and appraised via a structured assessment of the literature. Databases searched included Medline, EMBASE, and the Cochrane Libraries. Recommendations in the guidelines were developed by the panel to prioritise clinically important care decisions. The strength of each recommendation was determined according to a balance between desirable and undesirable consequences of alternative management strategies, the quality of the evidence (including the certainty of estimates), and the nature and variability of patient values and preferences.

**Results:** Key recommendations emphasise the importance of thorough diagnosis, treatment, and follow-up for patients with the conditions outlines. The guidelines stress the importance of a multidisciplinary approach to treatment and the importance of shared decision-making with patients and their guardians/caregivers. The summary provides evidence-based standards for management of undescended testes, testicular tumours in prepubertal boys, acute scrotum, hypospadias, congenital penile curvature, urinary tract infections, daytime lower urinary tract conditions, urachal remnants, and transitional urology.

**Conclusions:** Part II of the 2024 EAU/ESPU guidelines provides updated guidance for evidence-based management of a number of paediatric urological conditions, with recommendations designed for effective integration into clinial practice.

***Abstract 38***

Claude

Word count: 261

**Background:** Male sexual dysfunction (MSD) frequently coexists with infertility, yet its management is often neglected in favor of reproductive interventions. The purpose of these guidelines is to identify the various types of MSD associated with infertility and to provide evidence-based recommendations and expert opinion on their management.

**Methods:** An initial exploratory analysis identified subtopics of MSD associated with infertility, which were formulated into provisional recommendations based on expert opinion. Multiple literature searches were performed using Medline and PubMed databases, up to June 2024, to identify pertinent studies. The available evidence was carefully reviewed to create summaries and reframe initial recommendations. Modified recommendations were subjected to multiple rounds of revision using a modified Delphi process until consensus was reached. Recommendations were further discussed and revised at the 5th International Consultation on Sexual Medicine (Madrid, June 2024) and graded as weak or strong using GRADE criteria.

**Results:** Eleven recommendations were developed. The prevalence of sexual dysfunction in infertile men ranged from 17.8% to 61.6%. Female sexual dysfunction prevalence ranged from 43% to 90%, and MSD from 6.7% to 75%. Erectile dysfunction prevalence in infertile men was 17.8% to 85.9%, with an odds ratio of 2.92 (95% CI 1.71–4.96). Premature ejaculation prevalence was 8%–31%. Anorgasmic anejaculation prevalence was approximately 1.5 per 1000 men. Unconsummated marriage constituted 4%–17% of sexual health clinic visits. Intracavernosal injection achieved consummation rates of 67.7%–91%.

**Conclusion:** These evidence-based recommendations provide clinicians with a structured framework for identifying and managing MSD in infertile couples, emphasizing multidisciplinary evaluation, individualized treatment, and integration of sexual health into fertility care.

***Abstract 39***

GPT5

Word count: 335

**Background:** To develop and evaluate predictive models using demographic and baseline symptom questionnaire data to identify pediatric patients with bowel and bladder dysfunction at risk of non-response to standard urotherapy.

**Methods:** This retrospective cohort study included children aged 5–10 years evaluated from 2020 to 2023 at a pediatric urology clinic. Eligible patients completed a validated 18-item bowel and bladder dysfunction questionnaire at baseline and follow-up, attended at least 2 visits, and received standard urotherapy alone. The primary outcome was non-response at first follow-up, defined as unchanged or worsening symptom score. Multivariable logistic regression and random forest models incorporated age, sex, concurrent stool softener therapy, and 12 candidate symptom variables, totaling 15 predictors. Logistic regression used 80/20 holdout validation; the random forest model used 500 trees with 80/20 holdout validation. Discrimination was assessed by area under the receiver operating characteristic curve.

**Results:** Among 123 patients, 92 (75%) were female, median age was 6 years (IQR 5, 8), median follow-up was 3 months (IQR 1, 4), 100 (81%) had private insurance, and 81 (66%) received concurrent stool softeners. Median baseline and follow-up symptom scores were 4 (IQR 3, 5) and 2 (IQR 1, 4), respectively. Complete improvement occurred in 26 (21%); cumulative moderate-or-better and minimal-or-better improvement occurred in 54 (44%) and 77 (62%), corresponding to exclusive moderate improvement in 28 (23%), minimal improvement in 23 (19%), and no improvement in 46 (38%). On multivariable logistic regression, older age (OR 1.45, 95% CI 1.09, 1.98; p = 0.01) and dysuria (OR 1.54, 95% CI 1.06, 2.37; p = 0.03) predicted response, whereas daytime incontinence predicted lower response odds (OR 0.67, 95% CI 0.46, 0.97; p = 0.04). Logistic regression and random forest achieved AUROCs of 0.67 and 0.71; at a 40% threshold, the random forest identified an additional 1 in 10 non-responders.

**Conclusion:** Demographic and symptom-based predictors derived from a standardized questionnaire identified children at risk of urotherapy non-response. Random forest showed modestly higher discrimination than logistic regression, while age, dysuria, and daytime incontinence were key predictors.

**Abstract 40**

Claude

Word count: 296

**Background:** To evaluate the association between prone versus supine positioning and perioperative and postoperative outcomes in patients undergoing suction mini-percutaneous nephrolithotomy (mini-PCNL).

**Methods:** Data were prospectively collected from the Suction Technology Utility in mini-PCNL Study (STUMPS) registry across 30 centers in 21 countries between March and November 2024. Adult patients aged ≥18 years with kidney stones undergoing suction mini-PCNL using a 14–22 Fr suction sheath were included. Patient demographics, operative parameters, stone-free status, and 30-day complications were recorded. Stone-free status was graded on postoperative CT imaging. Multivariable logistic regression, adjusted for stone volume, puncture modality, tract dilation method, mechanical lithotripsy, and sheath size, was used to assess the effect of prone positioning on grade A stone-free status and overall complications.

**Results:** A total of 1,534 patients were analyzed; 653 (43%) underwent surgery in the prone position and 881 (57%) in the supine position. Grade A stone-free status was achieved in 85% of prone and 81% of supine cases (p = 0.052). Blood transfusion (Clavien grade 2) was significantly higher in the prone group (2.8% vs 0%; p < 0.001). Renal pelvic perforation was more frequent in the prone group (2.8% vs 0.23%; p < 0.001). Pneumothorax was exclusively observed in the prone group (1.5% vs 0%; p < 0.001). On multivariable analysis, prone positioning was not significantly associated with grade A stone-free status (aOR 0.92, 95% CI 0.66–1.29; p = 0.6) or overall complications (aOR 0.87, 95% CI 0.59–1.28; p = 0.5).

**Conclusion:** Suction mini-PCNL achieves comparable stone-free rates and overall complication rates in both prone and supine positions. However, prone positioning was associated with higher rates of specific complications including blood transfusion, renal pelvic perforation, and pneumothorax. The observational study design precludes definitive causal conclusions, and patient-specific factors should guide positional selection.

***Abstract 41***

Gemini

Word count: 249

**Background:** This review highlights existing and evolving medical therapeutics for managing male erectile dysfunction (ED), focusing on recent advancements in diagnostic pathways, pharmacotherapy, and treatment recommendations for specific patient populations.

**Methods:** A committee of experts conducted an extensive literature review and used a modified Delphi method to develop evidence-based recommendations. These preliminary guidelines were presented to global experts at the International Consultation of Sexual Medicine (ICSM) in July 2024 to achieve final consensus on management algorithms.

**Results:** Diabetes prevalence in ED is 66% for type 2 and 37.5% for type 1. Meta-analysis shows men with ED have a 45% higher risk of cardiovascular disease (RR 1.45). Regarding PDE5 inhibitors, sildenafil (50 mg) improved IIEF scores in 85% of hemodialysis patients compared to 9.5% for placebo. For spinal cord injuries, 85% of patients on sildenafil, 74% on vardenafil, and 72% on tadalafil achieved erections adequate for penetration. Daily tadalafil (5 mg) showed a 1.24-point improvement in IIEF. In Parkinson’s disease, sildenafil (25–100 mg) showed efficacy for penetration as high as 85%. Alprostadil injections enabled sexual intercourse after 94% of injections, with 87% satisfaction. Trimix salvages 50%–62% of PDE5i non-responders and 50% of those failing Bimix. Success rates for papaverine reach 80%, though corporal fibrosis occurs in 5%–30% and priapism in 3%–19%.

**Conclusion:** Oral PDE5 inhibitors remain the primary pharmacotherapy for most men. Advanced treatments like intracavernosal injections provide high success rates for refractory cases, though clinician selection must account for specific comorbidities and potential side effects.

***Abstract 42***

GPT5

Word count: 252

**Background:** To compare Aquablation and laser enucleation (LEP) for efficacy and safety in lower urinary tract symptoms with prostates of 80–180 ml.

**Methods:** WATER III was a trial with randomized/nonrandomized cohorts. Men aged 45–80 years with IPSS ≥8 underwent Aquablation or LEP. Primary endpoints were 3-month IPSS change and grade ≥2 or persistent grade 1 adverse events. Bayesian models were used.

**Results:** Of 202 patients, 74 were randomized and 128 nonrandomized; 98 underwent Aquablation and 88 LEP, and 170 completed follow-up. Age was 65.2±6.8 versus 67.5±6.6 years (p=0.02); prostate volume was 105.3±20.0 versus 108.7±24.0 ml, IPSS 20.7±5.7 versus 21.5±6.4, Qmax 10.7±4.5 versus 10.8±4.2 ml/s, and postvoid residual 101.2±81.8 versus 113.6±98.4 ml. IPSS improvement was 12.9±6.9 versus 13.1±7.5, with posterior probability of noninferiority >0.999, posterior mean difference 0.93, and 95% credible interval −1.48 to 3.53; 3-month IPSS was 7.9±5.1 versus 8.2±5.5. Grade ≥2/persistent grade 1 complications occurred in 40.8% versus 56.8% (difference −9.4%; 95% credible interval −31.8% to 12.9%; noninferiority probability 0.952); grade ≥3 adverse events occurred in 8.2% versus 5.7%, urinary tract infection in 28.6% versus 23.9%, any incontinence in 18.8% versus 38.9% (p<0.05), stress incontinence in 0% versus 9.3% (p<0.05), and persistent de novo ejaculatory dysfunction in 14.8% versus 77.1% (p<0.0001). At 3 months, Qmax was 19.8±10.6 versus 27.5±11.3 ml/s (p<0.0001), postvoid residual 48±66 versus 39±38 ml, and prostate volume 51 versus 26 ml.

**Conclusion:** Aquablation achieved noninferior improvement versus LEP, with lower ejaculatory dysfunction and stress urinary incontinence, whereas LEP yielded greater Qmax improvement and lower prostate volume.

***Abstract 43***

Human

Word count: 313

**Background:** Urinary incontinence is defined as ”the complaint of involuntary loss of urine” and affects approximately 40% of women. It is associated with morbidity and decrease in quality of life. Despite its prevalence, there are several challenges associated with its management and there is a need for nonsurgical treatment alternatives. This systematic review aimed to assess the effectiveness of intravaginal electrical stimulation in improving patient outcomes and quality of life.

**Methods**: Article titles and abstracts were independently double-screened. The full text of eligible articles were retrieved and assessed for eligibility by at least 2 independent authors. Bias was assessed by 2 authors (R.R.W. and M.K.) using the Cochrane Risk of Bias Tool 2.0. Disputes were resolved through consensus. As a meta-analysis could not be performed due to trial quality limitations and heterogeneity, findings are presented in a narrative tabular form.

**Results**: Among 187 studies identified, 32 randomized controlled trials (17.1%) were eligible for inclusion. When reporting specific outcomes, 6/8 (75%) randomized controlled trials reported significant within-group reductions in the number of pads used for intravaginal electrical stimulation groups; 10/15 (66.7%) trials reported significant within-group reductions in pad weight for intravaginal electrical stimulation groups; and 8/14 (57.1%) trials reported significantly reduced leakage frequencies with significant differences between intravaginal electrical stimulation and passive treatment groups. Other bladder diary measurement findings were mixed. Increased pelvic floor muscle contraction strength, increased maximum cystometric capacity, decreased detrusor overactivity, and increased quality of life were observed, although the evidence is of low quality and lacks robustness. In the setting of randomized controlled trials, intravaginal electrical stimulation exhibited moderate-to-high adherence rates and a low risk and severity of adverse events.

**Conclusion**: Intravaginal electrical stimulation may be a viable treatment for urinary incontinence. However, the present evidence base is of low quality to draw sufficient conclusions about its efficacy. Further multicentre, adequately powered and methodologically sound randomized controlled trials are needed.

***Abstract 44:***

Human

Word count: 291

**Background:** Achieving the highest possible stone-free rate is the primary goal of kidney stone surgery, yet standard flexible ureterorenoscopy often leaves small residual fragments that predispose to recurrence. The hydrogel method using mediNiK® was developed to embed and extract even the smallest fragments with conventional baskets. This study aimed to evaluate the safety and feasibility—defined as the ability to extract fragments < 1 mm—of stone extraction during flexible ureterorenoscopy with or without the hydrogel method.

**Methods:** This prospective, randomized, multicenter proof-of-concept trial included patients > 18 years with kidney stones > 8 mm and no anatomical abnormalities. Patients were randomized to flexible ureterorenoscopy + hydrogel (Group 1) or flexible ureterorenoscopy (Group 2) alone. After laser lithotripsy, fragments were retrieved either embedded in hydrogel or individually, and categorized by size (< 0.5 mm, 0.5–1.0 mm, > 1.0 mm). Explorative statistical analyses included Mann-Whitney U, Student’s t-test, and Chi-square test. Adverse events were monitored intraoperatively and during a 6-week follow-up.

**Results:** Of 65 screened patients, 40 were analysed (Group 1: n = 23; Group 2: n = 17). The hydrogel method significantly retrieved more fragments < 1 mm (1716 vs. 209) and > 1 mm (310 vs. 118). On a per-patient level, more < 1 mm fragments were removed in Group 1 (median 7 [IQR 21] vs. 0 [2], p < 0.003). Surgery duration was longer in Group 1 (80 [28] vs. 62 [20] minutes, p = 0.02). No serious adverse events were reported.

**Conclusions:** The hydrogel method was a safe and feasible addition to fURS, allowing improved retrieval of even the smallest fragments without added risk. The main limitations are the small sample size and absence of long-term stone-free and recurrence data, underscoring the need for larger confirmatory studies.

***Abstract 45:***

Gemini

Word count: 268

**Background:** The study aimed to evaluate the association between prone versus supine positioning and perioperative outcomes of suction-assisted mini-percutaneous nephrolithotomy (mini-PCNL). Researchers sought to provide comparative effectiveness data regarding stone clearance and complications for clinicians performing these procedures.

**Methods:** A prospective analysis of 1534 adult patients across 30 centers in 21 countries was conducted between March and November 2024. Patients with kidney stones underwent suction mini-PCNL using 14–22 Fr sheaths. The primary outcomes included 30-day stone-free rates (SFR), assessed via low-dose CT, and complication rates classified by the Clavien system. Multivariable regression adjusted for stone volume, lithotripsy modality, and sheath size.

**Results:** Of 1534 patients, 653 (43%) were prone and 881 (57%) supine. Prone patients had lower BMI (25.0 vs 27.2 kg/m², p<0.001) and smaller stones (1.8 vs 2.1 cm, p<0.001). Prone positioning was associated with higher rates of blood transfusion (2.8% vs 0%, p<0.001), renal pelvic perforation (2.8% vs 0.23%, p<0.001), and pneumothorax (1.5% vs 0%, p<0.001). Grade A SFR was 85% for prone and 81% for supine (p=0.052). Multivariable analysis showed positioning was not significantly associated with grade A SFR (aOR 0.92, 95% CI 0.66–1.29, p=0.6) or overall complications (aOR 0.87, 95% CI 0.59–1.28, p=0.5). Median operation time (47 vs 44 min, p=0.6) and hospital stay (2.0 vs 3.0 days, p=0.16) were similar.

**Conclusion:** Suction mini-PCNL achieves comparable stone-free rates and overall safety in both prone and supine positions. Although prone positioning was linked to higher specific risks like transfusion and pleural injury, overall complication rates did not differ significantly after adjustment. Surgeons should select positions based on patient characteristics and institutional expertise.

***Abstract 46***

Human

Word count: 283

**Background:** Oligometastatic prostate cancer (omPC) is characterized by limited metastases. We hypothesized that metastasis-directed therapy (MDT) to all sites of omPC combined with androgen deprivation therapy (ADT) would improve clinical outcomes.

**Methods:** In the multicenter phase 2 EXTEND trial, patients with omPC were randomized 1:1 to ADT versus MDT + ADT in two independently powered and randomized baskets, one using intermittent ADT and one using continuous ADT. The primary endpoint was progression-free survival (PFS). The secondary endpoints included radiologic PFS (rPFS) and castration resistance–free survival (CRFS). Here, the primary results of the continuous ADT basket, the combined analysis of both baskets, and translational immune correlatives are reported.

**Results:** From September 2018 through August 2022, 174 patients were randomized and were eligible for the primary analysis. In the continuous ADT bas- ket (N = 87), the median PFS was 47 mo with MDT + ADT versus 22 mo with ADT (hazard ratio [HR], 0.50; one-sided p = 0.036). In the combined analysis, the median PFS was 36 mo with MDT + ADT versus 17 mo with ADT (HR, 0.45; p < 0.001). Radiologic PFS and CRFS were also superior with MDT + ADT. Durable clinical responses after MDT + ADT were associated with systemic Th1-polarizing cytokine upregulation and CD8+ T-cell proliferation. Compared with ADT, MDT + ADT induced greater systemic immune activation, including T-cell receptor expansion/contraction, which we also observed in the independent ORIOLE trial of MDT. The greatest PFS benefit after MDT + ADT was observed in patients with systemic T-cell receptor expansion/contraction.

**Conclusions:** MDT + ADT improves PFS compared with ADT in omPC patients, meriting phase 3 confirmation. Hypothesis-generating immune responses warrant mechanistic validation and future trials with T-cell–targeted immunotherapies.

***Abstract 47***

Claude

Word count: 280

**Background:** The European Association of Urology/European Society of Paediatric Urology guidelines aim to provide evidence-based recommendations for the management of common urological conditions in children, including undescended testes, prepubertal testicular tumours, acute scrotum, hypospadias, congenital penile curvature, urinary tract infections, daytime lower urinary tract conditions, urachal remnants, and transitional urology.

**Methods:** New and relevant evidence was identified, collated, and appraised via a structured assessment of the literature. A broad and comprehensive scoping exercise covering all included topics was performed. Recommendations were developed by the panel to prioritise clinically important care decisions, with the strength of each recommendation determined by the balance between desirable and undesirable consequences, the quality of evidence, and the variability of patient values and preferences.

**Results:** Undescended testes affect 1.0–4.6% of full-term and 1.1–45% of preterm neonates, with surgical correction recommended by 12 months and no later than 18 months of age. Prepubertal testicular tumours occur at 0.5 to 2 per 100,000 children, with over 90% presenting as painless scrotal masses and ultrasound detection approaching 100%. Hypospadias primary repair success rates are 85–90% for distal and 32–86% for proximal cases, with fistula rates of 5–50% and meatal stenosis in 5–15%. Congenital penile curvature occurs in 0.6–5% of births, with surgical correction success rates of 90–100%. Daytime lower urinary tract conditions have a prevalence of 2–25%. Testicular torsion requires intervention within 4–6 hours to prevent irreversible necrosis.

**Conclusion:** These updated guidelines provide comprehensive, evidence-based recommendations for managing paediatric urological conditions, emphasizing early intervention for undescended testes, testis-sparing surgery for prepubertal tumours, individualized hypospadias repair, and multimodal management of lower urinary tract conditions, with structured transition to adult care.

***Abstract 48***

Claude

Word count: 293

**Background:** Erectile dysfunction (ED) remains a prevalent condition with significant impact on quality of life, yet therapeutic management continues to evolve. This chapter of the International Consensus of Sexual Medicine (ICSM) critically evaluates existing and emerging medical therapeutics for male ED, synthesizing current evidence and expert consensus to provide comprehensive, evidence-based recommendations for clinical practice.

**Methods:** A committee of experts and thought leaders in ED management was assembled to develop evidence-based recommendations for Committee 20: Sexual Rehabilitation after Treatment for Prostate Cancer. An extensive literature review was conducted focusing on recent advancements in diagnosis and treatment. Preliminary recommendations were developed through iterative committee discussions, then presented at a dedicated ICSM session in Madrid, July 2024. The session included open discussions and consensus-building exercises. A modified Delphi method was used for finalizing recommendations.

**Results:** The committee identified phosphodiesterase-5 inhibitors (PDE5i) as the cornerstone of first-line oral pharmacotherapy, noting a 25%-35% placebo response rate in ED drug trials and dropout rates of up to 50% with PDE5i. For intracavernosal injection therapy, alprostadil at 10-20 mcg achieved penetration hardness erections in 80% of patients, with overall long-term satisfaction rates of 78%. Among combination injection formulations, Bimix demonstrated 94% overall efficacy, while Trimix showed ability to salvage 50%-62% of PDE5i non-responders. Novel agents including bremelanotide (PT-141) achieved base rigidity of 80% or greater at 20 mg doses in Phase 2A trials. Botulinum neurotoxin A (100 units intracavernosal) enabled sufficient erection for vaginal penetration in 53% of treated patients versus the control group, with statistically significant differences favoring BTX-100 by month six (P < .01).

**Conclusion:** Current evidence supports a stepwise, individualized approach to ED management, with PDE5i as first-line therapy, intracavernosal injections as effective second-line options, and several novel agents demonstrating promising early-phase results warranting further investigation.

***Abstract 49***

Gemini

Word count: 254

**Background:** To evaluate the activity and safety of neoadjuvant TAR-200, an intravesical gemcitabine-releasing system, combined with systemic cetrelimab or cetrelimab monotherapy in patients with muscle-invasive bladder cancer who are ineligible for or decline cisplatin-based neoadjuvant chemotherapy before radical cystectomy.

**Methods:** In the SunRiSE-4 phase 2 trial, patients with stage cT2–cT4aN0M0 muscle-invasive bladder cancer were randomized 5:3 to receive neoadjuvant TAR-200 plus cetrelimab or cetrelimab monotherapy. Treatment consisted of four 21-day cycles. TAR-200 (225 mg gemcitabine) was placed intravesically every 21 days, and cetrelimab (360 mg) was administered intravenously every 21 days. The primary endpoint was centrally confirmed pathological complete response (ypT0N0). Statistical analysis was descriptive; no formal hypothesis testing was planned for this interim analysis.

**Results:** The safety population included 120 patients (mean age 70.7 years; 85% male; 81% cT2). The efficacy-evaluable population comprised 53 patients in the combination cohort and 31 in the monotherapy cohort. In the TAR-200 plus cetrelimab cohort, the pathological complete response rate was 42% (95% CI 28–56) and the pathological overall response rate was 60% (95% CI 46–74). In the cetrelimab monotherapy cohort, these rates were 23% (95% CI 10–41) and 35% (95% CI 19–55), respectively. Grade 3 or worse treatment-related adverse events occurred in 11% of the combination cohort and 5% of the monotherapy cohort. One treatment-related death was reported.

**Conclusion:** Neoadjuvant TAR-200 plus cetrelimab showed a benefit in pathological complete response compared with cetrelimab monotherapy in patients with muscle-invasive bladder cancer. These interim results support continued investigation of this localized treatment combination.

***Abstract 50***

GPT5

Word count: 337

**Background:** To evaluate oncological and functional outcomes of three-dimensional artificial intelligence–driven augmented reality robot-assisted radical prostatectomy versus two-dimensional cognitive MRI-guided surgery in patients with preoperative MRI suspicious for bulging or extracapsular extension undergoing nerve-sparing intervention.

**Methods:** This multicenter prospective randomized controlled trial allocated patients in a 1:2 ratio to 3D-AR or MRI-guided surgery. Adults aged ≥40 years with monolateral localized or locally advanced prostate cancer and PIRADS version 2 lesions suspicious for bulging or extracapsular extension were enrolled from July 2022 to February 2025. The primary endpoint was positive surgical margin rate. Secondary endpoints included biochemical recurrence, biochemical recurrence-free survival, local/distant recurrence, sexual potency, and urinary continence. A post hoc analysis evaluated positive excisional biopsies and positive margins at preserved neurovascular bundles.

**Results:** Of 138 assessed patients, 136 were randomized; 49 in the 3D-AR group and 84 in the MRI group received the allocated intervention, and 82 reached 12 months of follow-up. Baseline values were age 68 versus 69 years, BMI 26 versus 26, PSA 7.8 versus 7.7 ng/ml, lesion volume 10 versus 6.1 mm, extracapsular extension 45% versus 32%, and bulging 55% versus 68% in the 3D-AR versus MRI groups. Operative time was 135 versus 127 minutes (p=0.08), biopsy time 4 versus 3 minutes (p=0.1), blood loss 442 versus 476 ml (p=0.3), postoperative complications 2.0% versus 8.3% (p=0.1), and positive surgical margins 47% versus 45% (p=0.8). Positive neurovascular bundle biopsy occurred in 52% versus 13% (p=0.001), and positive margins on preserved bundles after excisional biopsy in 22% versus 39% (p=0.047). At 12 months, social continence was 94% versus 75% (p=0.03), total continence 91% versus 72% (p=0.03; OR 0.9, 95% CI 0.4–2.0), potency 41% versus 40% (p=0.2), biochemical recurrence 18% versus 15% (p=0.7), and postoperative radiotherapy 18% versus 35% (p=0.046).

**Conclusion:** AI-driven 3D-AR guidance during nerve-sparing robot-assisted radical prostatectomy was associated with lower positive margins at preserved neurovascular bundles, higher 12-month continence rates, and less postoperative radiotherapy than 2D MRI-guided surgery, while overall positive surgical margins, potency recovery, and biochemical recurrence were similar between groups.

***Abstract 51***

Gemini

Word count: 242

**Background:** To develop a predictive model using demographic and baseline questionnaire-derived data to identify pediatric patients at high risk for early non-response to urotherapy for bowel and bladder dysfunction (BBD).

**Methods:** This retrospective cohort study evaluated 123 pediatric patients aged 5–10 years at a single institution between 2020 and 2023. Data including age, sex, and 15 symptom-based predictors from a validated 18-question BBD questionnaire were collected via electronic medical record review. Non-response was defined as an unchanged or worsening follow-up symptom score. Multivariable logistic regression and random forest models were developed using an 80/20 holdout validation method to predict treatment failure at the first follow-up visit.

**Results:** The cohort had a median age of 6 years (IQR 5, 8), was 75% female (n=92), and 66% (n=81) used stool softeners. At a median 3-month follow-up, 38% (n=46) of patients showed no improvement. Logistic regression (AUROC 0.67) identified older age (OR 1.45, 95% CI 1.09, 1.98; p=0.01) and dysuria (OR 1.54, 95% CI 1.06, 2.37; p=0.03) as predictors of response, while daytime incontinence (OR 0.67, 95% CI 0.46, 0.97; p=0.04) predicted non-response. The random forest model achieved an AUROC of 0.71, identifying daytime incontinence, age, and dysuria as the most significant features.

**Conclusion:** Demographic and symptom-based predictors can modestly enhance the prediction of urotherapy non-response in pediatric BBD. While machine learning models provide improved risk stratification over traditional methods, further external validation is required before widespread clinical implementation to facilitate timely transitions to

***Abstract 52:***

Gemini

Word count: 249

**Background:** The EXTEND phase 2 randomized basket trial investigated whether adding metastasis-directed therapy (MDT) to standard-of-care systemic therapy improves outcomes for patients with oligometastatic prostate cancer. Investigators also evaluated whether clinical improvements are associated with systemic antitumor immune responses induced by MDT.

**Methods:** Patients with one to five metastases were randomized 1:1 to receive MDT plus intermittent or continuous androgen deprivation therapy (ADT) or ADT alone. The primary endpoint was progression-free survival (PFS). Secondary endpoints included radiologic PFS (rPFS) and castration resistance-free survival (CRFS). Longitudinal immune profiling was performed using flow cytometry and T-cell receptor (TCR) sequencing to assess systemic immune modulation.

**Results:** Among 174 evaluable patients, 133 had hormone-sensitive disease and 41 had oligometastatic castration-resistant disease. MDT plus ADT significantly improved median PFS to 36 months compared to 17 months with ADT alone (HR 0.45, 95% CI 0.30–0.69, p<0.001). Significant improvements were also observed for rPFS (HR 0.63, 95% CI 0.40–0.97, p=0.04) and CRFS (HR 0.40, 95% CI 0.19–0.82, p=0.01). TCR repertoire modulation, defined as both expansion and contraction of TCR clones, was significantly greater after MDT plus ADT (p<0.001) and associated with improved PFS (HR 0.21, 95% CI 0.06–0.76, p=0.02). No grade 4 or 5 toxicities occurred after MDT.

**Conclusion:** Adding MDT to ADT for oligometastatic prostate cancer significantly improves PFS, rPFS, and CRFS, delaying progression to lethal disease. These clinical benefits are mediated in part by MDT-induced systemic antitumor immunity, supporting the use of TCR modulation as a biomarker for clinical outcomes.

***Abstract 53***

Human

Word count: 346

**Background:** Erectile dysfunction (ED) is a prevalent condition with multifactorial etiology and significant impact on men’s quality of life. Although standard therapies—such as phosphodiesterase type 5 inhibitors, vacuum devices, and penile prostheses—offer symptomatic relief, they do not address the underlying pathophysiology. Platelet-rich plasma (PRP), an autologous concentration of platelets and growth factors, has emerged as a potential regenerative treatment that may promote neurovascular repair and restore erectile function. To evaluate the efficacy and safety of PRP monotherapy for the treatment of erectile dysfunction through a systematic review and meta-analysis of randomized controlled trials (RCTs).

**Methods:** Following PRISMA guidelines, a comprehensive search of PubMed, EMBASE, Google Scholar, Cochrane Library, Scopus, and Web of Science was performed for RCTs assessing intracavernosal PRP injections in men with ED. Studies combining PRP with other regenerative therapies were excluded. Data were synthesized using a random-effects model to generate pooled standardized mean differences (SMDs) in International Index of Erectile Function (IIEF) scores at 1, 3, and 6 months. Risk of bias was assessed using the Cochrane RoB 2 tool, and certainty of evidence was evaluated using the Grading of Recommendations Assessment, Development and Evaluation (GRADE) framework.

**Results:** Seven RCTs including 512 participants were analyzed. PRP injection volumes ranged from 5 to 10 mL across 2-4 sessions. At 1 month, pooled SMD in IIEF was 0.28 (95% CI−0.11 to 0.67, P= .16); at 3 months, it was 0.29 (95% CI−0.08 to 0.67, P <.05); and at 6 months, it was 0.36 (95% CI−0.09 to 0.81, P= .12). Heterogeneity was high (I2 =74%-83%) across time points. Subgroup analyses showed no significant difference by risk of bias. PRP was well tolerated, with only isolated mild adverse events (hematoma, plaque formation) reported.

**Conclusion:** Current evidence does not support a consistent, clinically meaningful improvement in erectile function with PRP monotherapy compared with placebo. Despite an encouraging mechanistic rationale and safety profile, the high heterogeneity, small sample sizes, and methodological limitations of existing RCTs limit confidence in efficacy. Larger, standardized, double-blind trials with rigorous protocols are needed to clarify the role of PRP in ED management.

***Abstract 54***

Human

Word count: 273

**Background:** Aquablation and laser enucleation of the prostate (LEP) are treatments for alleviation of lower urinary tract symptoms (LUTS) that have not yet been directly compared in a prospective randomized trial. This study was designed to evaluate these treatments in terms of LUTS improvement and safety in men with large prostates.

**Methods:** WATER III is an investigator-initiated, international, multicenter, nonblinded, prospective noninferiority trial that includes randomized and nonrandomized partici- pants. Eligible patients had moderate to severe LUTS and a large prostate volume (80– 180 ml). The primary efficacy endpoint was the change in International Prostate Symptom Score (IPSS) from baseline to 3 mo. The primary safety endpoint was the inci- dence of Clavien-Dindo (CD) grade 2 or persistent CD grade 1 complications that had not resolved by 3 mo. Bayesian analyses were used to assess noninferiority.

**Results:** A total of 202 men were enrolled in the study, of whom 186 underwent surgery (98 Aquablation, 88 LEP). At 3 mo, data were available for 170 patients, including 66 randomized and 104 nonrandomized men. Both treatmentsshowed similar mean IPSS improvement at 3 mo: 12.9 ± 6.9 with Aquablation versus 13.1 ± 7.5 with LEP, with an estimated difference of 0.93 (95% credible interval [CrI] 1.48 to 3.53) and noninferiority probability of >0.999. The incidence of CD grade 2/ persistent grade 1 complications was 40.8% in the Aquablation group versus 56.8% in the LEP, with an estimated difference of 9.4% (95%CrI 31.8% to 12.9%; noninferiority probability 0.952). Retrograde ejaculation was less frequent after Aquablation (14.8% vs 77.1%; p < 0.001). Persistent stress urinary incontinence (SUI) was absent following Aquablation versus 9.3% after LEP (p < 0.05).

***Abstract 55***

GPT5

Word count: 318

**Background:** To develop evidence- and consensus-based recommendations for the clinical management of male sexual dysfunction in the setting of infertility. Infertility affects around 10% of couples and 12% of men, and sexual dysfunction in infertile men has been reported in 17.8%–61.6%. Because male sexual dysfunction may either contribute to infertility or arise during infertility evaluation and treatment, this review sought to define relevant clinical domains and provide practical management guidance.

**Methods:** An exploratory analysis identified subtopics linking male sexual dysfunction and infertility and generated provisional recommendations based on expert opinion. Focused literature searches relevant to each subtopic were performed using Medline and PubMed up to June 2024, and evidence summaries were used to revise the initial statements. Recommendations then underwent multiple rounds of modified Delphi discussion and were finalized at the 5th International Consultation on Sexual Medicine held in Madrid in June 2024, with grading categorized as strong, weak, or expert opinion.

**Results:** Reported prevalence estimates showed female sexual dysfunction in infertile couples ranging from 43% to 90%, male sexual dysfunction ranging from 48%–58%, and in another review 6.7%–75%. A meta-analysis comparing 2276 infertile men with 1492 fertile men found higher odds of erectile dysfunction in infertile men (OR 2.92, 95% CI 1.71–4.96), with erectile dysfunction rates ranging from 17.8% to 85.9%. Unconsummated marriage accounted for 4%–17% of sexual health clinic visits; reported contributors included vaginismus in 8.4%–81%, erectile dysfunction in 10.5%–61%, combined male and female factors in 16.6%–26%, performance anxiety in 4.1%–52.9%, and premature ejaculation in 0.5%–52%. Delayed ejaculation prevalence was estimated at 1.5/1000, and premature ejaculation prevalence ranged from 8% to 31%.

**Conclusion:** Male sexual dysfunction commonly coexists with infertility and warrants structured assessment and targeted management during fertility care. These consensus recommendations support detailed sexual history-taking, focused examination, multidisciplinary evaluation, and fertility-conscious treatment selection to address erectile, ejaculatory, hormonal, lubricant-related, and lifestyle-associated contributors to infertility.

***Abstract 56***

Claude

Word count: 275

**Background:** To evaluate the activity and safety of neoadjuvant TAR-200, an intravesical gemcitabine-releasing system, plus cetrelimab versus cetrelimab monotherapy in patients with muscle-invasive bladder cancer who are ineligible for or decline neoadjuvant cisplatin-based chemotherapy.

**Methods:** SunRISe-4 is an ongoing, randomized, open-label, phase 2 trial conducted at 109 centers across ten countries. Patients with histologically confirmed muscle-invasive bladder cancer (stage cT2–cT4 N0M0) scheduled for radical cystectomy were randomly assigned (5:3) to TAR-200 plus cetrelimab or cetrelimab monotherapy. TAR-200 (225 mg gemcitabine) was placed intravesically every 21 days for four cycles. Cetrelimab (360 mg) was administered intravenously every 21 days for four cycles. The primary endpoint was centrally confirmed pathological complete response (ypT0N0). A key exploratory endpoint was pathological overall response (≤ypT1N0). Results of the second prespecified interim analysis are reported.

**Results:** Of 196 patients assessed, 122 were randomly assigned (TAR-200 plus cetrelimab, n=80; cetrelimab monotherapy, n=42). The efficacy-evaluable population included 53 and 31 patients, respectively. Mean age was 70.7 years (SD 7.9); 86% were male and 68% were White. The pathological complete response rate was 42% (22/53; 95% CI 28–56) in the combination cohort versus 23% (7/31; 95% CI 10–41) with monotherapy. Pathological overall response rates were 60% (32/53; 95% CI 46–74) and 35% (11/31; 95% CI 19–55), respectively. Treatment-related adverse events occurred in 57 (72%) and 18 (44%) patients, respectively. Grade 3 or worse treatment-related adverse events occurred in 9 (11%) and 2 (5%) patients, respectively.

**Conclusion:** Neoadjuvant TAR-200 plus cetrelimab demonstrated higher pathological complete and overall response rates compared with cetrelimab monotherapy, with a manageable safety profile in patients with muscle-invasive bladder cancer ineligible for or declining cisplatin-based chemotherapy.

***Abstract 57***

Gemini

Word count: 260

**Background:** This systematic review evaluated the clinical utility of intravaginal electrical stimulation (IVES) as a nonsurgical intervention for women with urinary incontinence (UI) to inform evidence-based practice recommendations. The review compared outcomes including urinary leakage, patient-reported measures, pelvic floor muscle (PFM) strength, and quality of life (QoL).

**Methods:** Conducted according to PRISMA guidelines, a systematic literature search was performed across PubMed, EMBASE, Ovid Medline, and IEEE Xplore through February 2025. Inclusion criteria were randomized controlled trials (RCTs) evaluating IVES in women with pelvic organ prolapse or UI compared to conservative treatments, placebo, or biofeedback. Risk of bias was assessed using the Cochrane Risk of Bias 2.0 tool.

**Results:** Thirty-two RCTs were included, representing 4,898 initially identified records. Of the 32 trials, 31 (96.9%) examined a general population and one (3.1%) focused on postpartum women. Regarding UI subtypes, 19 trials investigated stress UI, 10 investigated mixed UI, and 14 investigated urgency UI. Ten trials assessed pad counts; 6 (75%) of 8 reporting trials found significant within-group reductions for IVES. For pad weight, 10 (66.7%) of 15 trials showed statistically significant reductions after IVES. Nineteen trials examined PFM strength, with 11 (57.9%) revealing significant changes in IVES groups. Adherence rates ranged between 61% and 100%. Risk of bias assessment showed high concerns in several domains, including 15 trials with high overall risk. Adverse events like vaginal irritation and discomfort were reported with low frequency.

**Conclusion:** IVES is a safe, acceptable intervention that may reduce urinary incontinence, though its superiority over other active treatments remains uncertain due to mixed evidence and low-quality data.

***Abstract 58***

GPT5

Word count: 293

**Background:** To summarize the 2024 EAU/ESPU Part II updates for pediatric urology addressing undescended testes, prepubertal testicular tumors, acute scrotum, hypospadias, congenital penile curvature, febrile urinary tract infection, daytime lower urinary tract conditions, urachal remnants, and transitional urology.

**Methods**: New and relevant evidence was identified, collated, and appraised through a structured literature assessment and broad scoping exercise. Recommendation strength was determined by evidence quality, balance of desirable and undesirable consequences, and patient values and preferences. This update focused on clinically important management decisions and incorporated new sections on rare childhood conditions and transitional urology.

**Results:** Undescended testis affects 1.0%–4.6% of full-term and 1.1%–45% of preterm neonates; treatment should start at 6 months and be completed by 12 months, or by 18 months at the latest. In nonpalpable testes, laparoscopy may show spermatic vessels entering the inguinal canal in 40%, an intra-abdominal testis in 40%, a peeping testis in 10%, and blind-ending vessels in 10%. Prepubertal testicular tumors have an incidence of 0.5–2 per 100 000 children, peak at 0–4 years, require 7.5–12.5 MHz Doppler ultrasonography, and showed postoperative recurrence of 5.8% (95% confidence interval 2.3%–14.1%) with benign findings in 70.9%. Testicular torsion requires action within 4–6 hours. For hypospadias, primary repair is usually performed at 6–18 months; curvature >30° typically warrants correction; severe repair may recur with curvature in 20% and stricture in 17%; distal outcomes are 85%–90% and proximal outcomes 32%–86%. Daytime lower urinary tract conditions affect 2%–25% of children, and transition discussions should begin at 12–16 years.

**Conclusion:** These updates emphasize timely intervention, risk-stratified imaging and surgery, structured functional assessment, and planned transition to adult care. Several recommendations remain limited by low-quality evidence, supporting individualized management and long-term follow-up.

***Abstract 59***

Human

Word count: 294

**Background:** Since 2016, >21 000 patients with prostate cancer (PC) used our personalized online decision aid in routine care in Germany. We analyzed the effects of this online decision aid for men with nonmetastatic PC in a randomized controlled trial.

**Methods:** In the randomized controlled EvEnt-PCA trial, 116 centers performed 1:1 allocation of 1115 patients with nonmetastatic PC to use an online decision aid (intervention = I) or a printed brochure (control = C). The primary outcome was treatment decision after 14 mo. The secondary endpoints were knowledge, acceptance, decisional conflict, physician-patient communication, anxiety and depression, decision regret, and quality of life.

**Results:** With complete data from 1000 patients, there was no difference between the two groups in terms of primary or secondary endpoints. In the intervention group, consultation time was shorter (I: 61 min vs C: 70 min, p = 0.02), and more patients provided a summary of their preparation (I: 76% vs C: 56%, p < 0.001). Life expectancy (I: 53% vs C: 47%, p = 0.03), radiotherapy (I: 90% vs C: 84%, p = 0.005), rehabilitation (I: 69% vs C: 62%, p = 0.02), and psycho-oncology (I: 21% vs C: 16%, p = 0.07) were discussed more frequently in the intervention group. Additionally, more counseling sessions with general practitioners (I: 43% vs C: 35%, p = 0.01) and radiotherapists (I: 24% vs C: 18%, p = 0.03) were held in the intervention group.

**Conclusions:** The use of the online decision aid was associated with structural improvements, for example, significantly shorter doctor-patient consultation time. Our findings suggest stronger guideline adherence in the counseling process and promotion of health literacy. Successful implementation of our online decision aid in routine care can provide an example for solving this key obstacle for shared decision making.

***Abstract 60***

GPT5

Word count: 252

**Background:** To evaluate randomized trial evidence on platelet-rich plasma (PRP) for erectile dysfunction.

**Methods:** This PRISMA review and meta-analysis, registered in PROSPERO (CRD42024605873), searched 6 databases for randomized trials of PRP in erectile dysfunction. Two reviewers screened studies, assessed RoB 2 risk of bias, and pooled IIEF outcomes with random-effects models.

**Results:** Seven trials with 512 participants were included. Sample sizes ranged from 52 to 120; PRP volumes, 5 to 10 cc; injection sessions, 2 to 4; and treatment intervals, 2 to 4 weeks. Of 309 records, 20 had full-text review; 5, 5, 2, and 1 were excluded for wrong study design, unavailable data, wrong intervention, and incomplete trial, leaving 7 studies. Response rates were 83% versus 17% at 6 months (P < .001), 72% versus 16% at 3 and 6 months (P < .001), and 69% versus 27% at 6 months. Pooled IIEF SMDs were 0.28 (95% CI, -0.11 to 0.67; P = .16; I2 = 77%; τ2 = 0.18; P < .01) at 1 month, 0.29 (95% CI, -0.08 to 0.67; I2 = 74%; τ2 = 0.16; P < .01) at 3 months, and 0.36 (95% CI, -0.09 to 0.81; P = .12; I2 = 83%; τ2 = 0.25; P < .01) at 6 months. Four trials had concerns for risk of bias, 3 had low risk, and adverse events were 1 hematoma and 1 plaque formation.

**Conclusion:** Current randomized evidence does not show a statistically significant benefit of PRP. Heterogeneity and study quality support larger trials with longer follow-up.
