# Supplementary material for: Confidence without consensus? Human versus AI abstracts in urology
Source: World J Urol. 2026 Oct 16;44(1):751. doi: 10.1007/s00345-026-06872-6 (PMC13675285; doi:10.1007/s00345-026-06872-6)
Supplement: Supplementary file 2 [file 345_2026_6872_MOESM2_ESM.docx]

**Supplementary Table S1.** Abstract characteristics by source.

| **Source** | **n** | **Word count, M (SD)** | **Range** | **Numerical values, M (SD)** | **Range** |
| --- | --- | --- | --- | --- | --- |
| Human | 15 | 315.9 (98.5) | 135–572 | 29.9 (23.5) | 2–90 |
| Claude Sonnet 4.6 | 15 | 282.5 (18.4) | 257–320 | 36.3 (11.5) | 21–57 |
| GPT-5.5 | 15 | 290.0 (46.7) | 250–377 | 65.8 (27.0) | 32–121 |
| Gemini 3 Flash | 15 | 264.8 (21.0) | 241–313 | 34.5 (10.6) | 21–54 |

*Note.* M = mean, SD = standard deviation. Word counts and numerical values were computed on the abstracts exactly as presented to reviewers. A numerical value was defined as any run of digits not embedded within a word, so that sample sizes, percentages, p values, and confidence-interval bounds each counted once. Mean length did not differ significantly between sources (repeated-measures analysis of variance blocked on source study, F(3,42) = 2.15, p = .109), whereas numerical density did (F(3,42) = 23.19, p < .001) and variance in length differed significantly (Fligner–Killeen χ²(3) = 10.93, p = .012). Per-abstract values are given in Supplementary Table S1b.

**Supplementary Table S1b.** Word count and number of numerical values for each abstract.

| **#** | **Words** | **Nums** | **Source** | **#** | **Words** | **Nums** | **Source** | **#** | **Words** | **Nums** | **Source** |
| --- | --- | --- | --- | --- | --- | --- | --- | --- | --- | --- | --- |
| 1 | 461 | 54 | Human | 21 | 272 | 31 | Claude | 41 | 245 | 28 | Gemini |
| 2 | 250 | 24 | Gemini | 22 | 240 | 12 | Human | 42 | 250 | 84 | GPT-5.5 |
| 3 | 250 | 76 | GPT-5.5 | 23 | 350 | 48 | Human | 43 | 313 | 16 | Human |
| 4 | 304 | 31 | Gemini | 24 | 306 | 37 | Claude | 44 | 288 | 35 | Human |
| 5 | 282 | 26 | Claude | 25 | 263 | 32 | GPT-5.5 | 45 | 265 | 46 | Gemini |
| 6 | 135 | 2 | Human | 26 | 268 | 49 | Claude | 46 | 281 | 16 | Human |
| 7 | 271 | 41 | Gemini | 27 | 250 | 82 | GPT-5.5 | 47 | 270 | 27 | Claude |
| 8 | 266 | 24 | Claude | 28 | 280 | 37 | Claude | 48 | 293 | 21 | Claude |
| 9 | 329 | 39 | Human | 29 | 252 | 46 | GPT-5.5 | 49 | 249 | 39 | Gemini |
| 10 | 312 | 55 | Claude | 30 | 264 | 54 | Gemini | 50 | 335 | 71 | GPT-5.5 |
| 11 | 313 | 21 | Gemini | 31 | 294 | 2 | Human | 51 | 241 | 36 | Gemini |
| 12 | 377 | 94 | GPT-5.5 | 32 | 250 | 41 | GPT-5.5 | 52 | 245 | 31 | Gemini |
| 13 | 257 | 53 | Gemini | 33 | 275 | 41 | Claude | 53 | 344 | 30 | Human |
| 14 | 281 | 38 | Gemini | 34 | 256 | 27 | Gemini | 54 | 273 | 38 | Human |
| 15 | 348 | 121 | GPT-5.5 | 35 | 309 | 31 | Human | 55 | 308 | 37 | GPT-5.5 |
| 16 | 274 | 36 | Claude | 36 | 271 | 28 | Gemini | 56 | 270 | 57 | Claude |
| 17 | 572 | 90 | Human | 37 | 255 | 2 | Human | 57 | 260 | 21 | Gemini |
| 18 | 350 | 100 | GPT-5.5 | 38 | 257 | 23 | Claude | 58 | 281 | 40 | GPT-5.5 |
| 19 | 320 | 46 | Claude | 39 | 334 | 64 | GPT-5.5 | 59 | 294 | 33 | Human |
| 20 | 250 | 37 | GPT-5.5 | 40 | 293 | 35 | Claude | 60 | 252 | 62 | GPT-5.5 |

*Note.* Abstracts are numbered in the order presented to reviewers. Words = total word count; Nums = number of numerical values, defined as in Supplementary Table S1. Source names are abbreviated: Claude = Claude Sonnet 4.6; Gemini = Gemini 3 Flash.
